# Gene signature of children with severe respiratory syncytial virus infection

**DOI:** 10.1038/s41390-020-01347-9

**Published:** 2021-01-28

**Authors:** Clyde Dapat, Satoru Kumaki, Hiroki Sakurai, Hidekazu Nishimura, Hannah Karen Mina Labayo, Michiko Okamoto, Mayuko Saito, Hitoshi Oshitani

**Affiliations:** 1 Department of Virology, Tohoku University Graduate School of Medicine, 2-1 Seiryo-machi, Aoba-ku, Sendai, 980-8575 Japan; 2 Department of Pediatrics, Sendai Medical Center, 11-12 Miyagino 2-chome, Miyagino-ku, Sendai, 983-8520 Japan; 3 Department of General Pediatrics, Miyagi Children’s Hospital, 3-17 Ochiai 4-chome, Aoba-ku, Sendai, 989-3126 Japan; 4 Virus Research Center, Sendai Medical Center, 11-12 Miyagino 2-chome, Miyagino-ku, Sendai, 983-8520 Japan

## Abstract

**Background:**

The limited treatment options for children with severe respiratory syncytial virus (RSV) infection highlights the need for a comprehensive understanding of the host cellular response during infection. We aimed to identify host genes that are associated with severe RSV disease and to identify drugs that can be repurposed for the treatment of severe RSV infection.

**Methods:**

We examined clinical data and blood samples from 37 hospitalized children (29 mild and 8 severe) with RSV infection. We tested RNA from blood samples using next-generation sequencing to profile global mRNA expression and identify cellular processes.

**Results:**

Retractions, decreased breath sounds, and tachypnea were associated with disease severity. We observed upregulation of genes related to neutrophil, inflammatory response, blood coagulation, and downregulation of genes related to T cell response in children with severe RSV. Using network-based approach, 43 drugs were identified that are predicted to interact with the gene products of these differentially expressed genes.

**Conclusions:**

These results suggest that the changes in the expression pattern in the innate and adaptive immune responses may be associated with RSV clinical severity. Compounds that target these cellular processes can be repositioned as candidate drugs in the treatment of severe RSV.

**Impact:**

- Neutrophil, inflammation, and blood coagulation genes are upregulated in children with severe RSV infection.
- Expression of T cell response genes are suppressed in cases of severe RSV.
- Genes identified in this study can contribute in understanding the pathogenesis of RSV disease severity.
- Drugs that target cellular processes associated with severe RSV can be repositioned as potential therapeutic options.

## Introduction

Acute respiratory tract infections due to viral pathogens are the major causes of illness and death in children worldwide. The leading cause of acute lower respiratory tract infection among children younger than 5 years of age is respiratory syncytial virus (RSV), which affects 33.1 million children and responsible for 3.2 million hospital admissions and 59,600 in-hospital deaths per year worldwide.^1^ Almost all children will get infected with RSV in their first year of life that is characterized by a spectrum of infection from mild respiratory symptoms to severe pneumonia and bronchiolitis, which affects 3.4 million children worldwide.^2^ Children with severe RSV require hospitalization and some would need intensive care.

To date, vaccine against RSV is under development and the treatment option for children with severe RSV is limited. This may be due to our incomplete understanding of the host cellular responses during RSV infection. One of the major difficulties in providing host-directed therapy is the lack of reliable diagnostic tools in the management of severe RSV. Several in vitro studies have been conducted using high throughput technologies to identify host factors that are affected during RSV infection.^3–5^ However, these studies utilized in vitro cell lines and laboratory-adapted strains of RSV, which may not reflect the clinical course of the disease. Studies were conducted using whole blood from children with acute respiratory infections to distinguish between mild and severe forms of pneumonia.^6–9^ These studies utilized various case definitions of severe RSV, which may be challenging to implement across different settings. The World Health Organization (WHO) working group proposed a case definition for severe and very severe RSV-associated lower respiratory tract illness (LRTI), which is based on clinical features that are objective and easy to understand including cough, difficulty breathing, and SpO_2_ measurements.^10^

Combining genomic data with clinical information may provide insight into the development of RSV disease severity in children. This study aimed to identify host genes in children that are associated with severe RSV-associated lower respiratory tract infection using the WHO case definition. This study also aimed to identify host genes that codes for proteins as potential drug targets, which will interact with drugs as possible treatment options for severe RSV infection.

## Methods

### Study design, setting, and participants

This was a prospective observational study of hospitalized children infected with RSV evaluating the host genes that were associated with severe disease. We enrolled children younger than 5 years old with coughing, sneezing, nasal discharge, wheezing, or breathing difficulties admitted at Sendai Medical Center and Miyagi Children’s Hospital in Sendai, Japan during the two RSV seasons from October 2017 to March 2019. We excluded children with known heart, lung, liver, or kidney disease, immunodeficiency, gestational age <36 weeks, received steroid treatment within 2 weeks or palivizumab treatment within 4 weeks. This study was approved by the Tohoku University Graduate School of Medicine Ethics Committee, Sendai Medical Center Ethics Committee, and Miyagi Children’s Hospital Ethics Committee. Written informed consent was obtained from the parent or guardian before participating in the study.

### Data and sample collection

Demographic and clinical information were collected upon admission using questionnaires and medical records. Nasal samples (nasopharyngeal swabs, nasal aspirate, or nasal wash) were collected upon admission and tested for RSV by the rapid diagnostic test kit. Peripheral whole blood (1 mL) was collected upon admission using PAXgene Blood RNA tubes (PreAnalytix GmbH, Hombrechtikon, Switzerland). Other clinical data including length of hospital stay, oxygen administration, chest X-ray, and blood count were collected after hospital discharge.

### Assessment of RSV severity

Severity of the disease was assessed based on the WHO case definition for severe and very severe RSV-associated lower respiratory tract infection.^10^ Respiratory tract illness (RTI) was defined as having cough and/or difficult breathing. LRTI was defined as having RTI (cough or difficulty breathing) and tachypnea (≥60 breaths per min for children <2 months, ≥50 breaths per min for children 2–11 months, and ≥40 breaths per min for children 12–23 months) or oxygen saturation (SpO_2_) value of <95%. Severe LRTI was defined as LRTI and lower chest wall indrawing or SpO_2_ < 93%. And very severe LRTI was defined as LRTI and inability to feed, failure to respond, or unconscious or SpO_2_ < 90%. In this study, children were classified as mild RSV if they presented any of the criteria under RTI or LRTI category and children were classified as severe RSV if they had severe or very severe LRTI. Tachycardia was defined as a heart rate of ≥180 beats/min in children <12 months and ≥140 beats/min in children ≥12 months.

### RSV detection and typing

Total RNA from nasal samples was extracted using QIAamp MinElute virus spin kit (Qiagen, Hilden, Germany) and cDNA was transcribed using Moloney murine leukemia virus reverse transcriptase and random primers (Invitrogen, Carlsbad, CA). Detection and typing of RSV by PCR and sequencing was performed as described previously.^11,12^

### RNA extraction and next-generation sequencing

Total RNA from blood samples was extracted using the MagMAX for Stabilized Blood Tubes RNA Isolation Kit (ThermoFisher Scientific, Vilnius, Lithuania). Quality control of isolated RNA was assessed using the Agilent RNA 6000 Pico Kit (Agilent Technologies, Waldbronn, Germany). Library preparation was performed using TruSeq Stranded Total RNA with Ribo-Zero Globin Kit (Illumina, San Diego, CA). Libraries were sequenced using the Illumina Hiseq 2500 (Illumina, San Diego, CA) at a target depth of about 20 million 51-nucleotide single-end reads per sample.

### Differential gene expression analysis

Next-generation sequencing (NGS) data were analyzed using the SeqBox system.^13^ Briefly, raw reads were trimmed using skewer,^14^ mapped to the hg38 human reference genome using STAR,^15^ and quantified with RSEM.^16^ PCAExplorer R package version 2.14.2 (ref. ^17^) was used for principal component analysis and heatmap visualizations on normalized and log-transformed sequence data by the variance stabilizing transformation method.^18^ Differential expression analysis was performed on un-normalized sequence count data by comparing the two groups (mild vs. severe RSV) after adjusting for age, sex, and batch by enrollment year and hospital using DESeq2 R package version 1.28.1.^19^ Results of DESeq2 analysis included *p* values, adjusted *p* values, and log2 fold changes with positive log fold change values indicate increased gene expression while negative values indicate decreased gene expression in the severe RSV group when compared to mild RSV group. The empirical Bayes method was used to adjust for nonbiological variation between batches as implemented in the surrogate variable analysis (SVA) R package version 3.36.0.^20^ False discovery rate (FDR) was calculated by applying the weighted Benjamin–Hochberg method for multiple hypothesis testing using the independent hypothesis weighting (IHW) software.^21^ A gene was considered differentially expressed if its FDR < 0.05. NGS data are deposited in the NCBI Gene Expression Omnibus (GEO accession number: GSE155925).

### Functional enrichment analysis

To determine the biological function of differentially expressed genes, modular transcriptome analysis was performed using the tmod R package version 0.44.^22^ GO database release version 2020-07 (refs. ^23,24^) and KEGG database release version 95.0 (ref. ^25^) were used in the functional enrichment analysis.

### Gene network analysis

Gene co-expression network was generated by querying the list of differentially expressed genes to the STRING database version 11.0 (ref. ^26^). Genes that are expressed only in blood were included in the analysis to minimize false positives. The reconstructed network was analyzed and visualized using Cytoscape software version 3.8.0.^27^ Highly connected nodes were considered as hubs and were identified using MCODE package version 1.6.1.^28^ Annotation of genes was determined using BiNGO package version 3.0.4.^29^ The list of differentially expressed genes was narrowed down to genes that belonged to a network hub and tmod module and fold change ≥2 or ≤−1.5.

### Drug–target network analysis

To identify drugs that can be repurposed for the treatment of severe RSV diseased, drug–target network analysis was performed. The drug–target network was generated by querying the list of genes that belonged to network hubs and tmod modules to the DrugBank database version 5.1.7.^30^ The network was analyzed and visualized using Cytoscape.^27^

### Statistical analysis

Demographic and clinical data were summarized using descriptive statistics. Distributions of categorical variables are shown as numbers with percentages per mild and severe RSV group. Continuous variables are reported as median with interquartile range (IQR). Fisher’s exact test was used to compare the differences in proportions and Mann–Whitney test was used to compare the differences in the distributions of median values between the two groups. A *p* value < 0.05 was considered statistically significant. The Benjamini–Hochberg method was used to correct for multiple testing.^31^ Statistical analyses were performed in R version 3.6.2.^32^

## Results

### Demographic and clinical characteristics

There were 38 children who tested positive by PCR for RSV during the study period. One child had a low RNA content in the blood sample and was excluded in the subsequent analyses. Of the remaining children, 29 (22 RTI and 7 LRTI) belonged to the mild RSV group while 8 (4 severe LRTI and 4 very severe LRTI) belonged to the severe RSV group (Table 1). Upon admission, children in the severe RSV group had higher proportion of retractions (62.5% vs. 6.9%, *p* = 0.002), decreased breath sounds (25% vs. 0%, *p* = 0.042), and tachypnea (62.5% vs. 18.5%, *p* = 0.027) than children in the mild RSV group. There were no significant differences with regard to age or sex between groups.

**Table 1:** Demographic and clinical characteristics of RSV patients classified by WHO LRTI.

Characteristics	Mild RSV (*n* = 29)	Severe RSV (*n* = 8)	*P* value^a^
Sex			
Female	13 (44.8)	5 (62.5)	
Male	16 (55.2)	3 (37.5)	0.447
Age in months, median [IQR]	12 (3–17)	18 (13–30)	0.364
0 to <12 months	16 (55.2)	2 (25.0)	
12 to <24 months	10 (34.5)	3 (37.5)	
24 to <36 months	2 (6.9)	1 (12.5)	
36 to <60 months	1 (3.4)	2 (25.0)	
Axillary temperature			
<38 °C	15 (51.7)	4 (50.0)	
≥38 °C	14 (48.3)	4 (50.0)	1.000
Wheeze			
No	20 (69.0)	4 (50.0)	
Yes	9 (31.0)	4 (50.0)	0.413
Retractions			
No	27 (93.1)	3 (37.5)	
Yes	2 (6.9)	5 (62.5)	0.002
Rales			
No	13 (44.8)	2 (25.0)	
Yes	16 (55.2)	6 (75.0)	0.431
Decreased breath sounds			
No	29 (100.0)	6 (75.0)	
Yes	0 (0.0)	2 (25.0)	0.042
Accessory muscle utilization			
No	28 (96.6)	6 (75.0)	
Yes	1 (3.4)	2 (25.0)	0.112
Tachypnea^b^			
No	22 (81.5)	3 (37.5)	
Yes	5 (18.5)	5 (62.5)	0.027
Tachycardia^c^			
No	20 (54.2)	5 (62.5)	
Yes	6 (45.8)	3 (37.5)	0.649
SpO_2_ %, median [IQR]	96 [95–97]	93 [90–95]	0.020
SpO_2_ < 93%			
No	29 (100.0)	4 (50.0)	
Yes	0 (0.0)	4 (50.0)	0.001
SpO_2_ < 90%			
No	29 (100.0)	6 (75.0)	
Yes	0 (0.0)	2 (25.0)	0.042
Oxygen administration			
No	16 (55.2)	3 (37.5)	
Yes	13 (44.8)	5 (62.5)	0.447
Duration of O_2_ administration, h	0 [0–33]	37 [0–108]	0.190
Length of hospitalization, days	4 (2–6)	6 (5–8)	0.220
Complete blood count results			
Red blood cells, 10^6^/μL	4.5 [4.3–4.9]	4.5 [4.3–4.7]	0.644
Hemoglobin, g/dL	11.5 [11.0–12.3]	11.3 [11.0–12.0]	0.956
Hematocrit, %	35.0 [32.1–37.0]	34.4 [33.7–35.8]	0.883
White blood cells, 10^3^/μL	9.4 [7.4–12.3]	10.8 [10.1–13.2]	0.086
Neutrophils, %	34.0 [25.3–42.5]	60.5 [48.5–68.8]	0.001
Lymphocytes, %	56.7 [46.6–60.6]	30.2 [24.1–42.7]	0.001
Monocytes, %	8.9 [7.5–10.9]	7.5 [6.8–8.0]	0.035
Platelets, 10^4^/μL	33.3 [28.3–42.6]	32.5 [24.1–34.8]	0.356
Chest X-ray findings			
Bronchial wall thickening			
No	27 (93.1)	8 (100.0)	
Yes	2 (6.9)	0 (0.0)	1.000
Atelectasis			
No	29 (100.0)	7 (87.5)	
Yes	0 (0.0)	1 (12.5)	0.216
Consolidation			
No	15 (51.7)	2 (25.0)	
Yes	14 (48.3)	6 (75.0)	0.246
Infiltration			
No	25 (61.5)	7 (87.5)	
Yes	4 (38.5)	1 (12.5)	1.000
Negative finding			
No	20 (69.0)	8 (100.0)	
Yes	9 (31.0)	0 (0.0)	0.159
RSV subgroup A			
No	10 (34.5)	2 (25.0)	
Yes	19 (65.5)	6 (75.0)	1.000
WHO LRTI classification			
RTI	22 (75.9)	0 (0.0)	
LRTI	7 (24.1)	0 (0.0)	
Severe LRTI	0 (0.0)	4 (50.0)	
Very severe LRTI	0 (0.0)	4 (50.0)	<0.001

Values reported are median [interquartile range, IQR] or *n* (%). *RTI* respiratory tract illness, *LRTI* lower respiratory tract illness.

^a^*P* values were calculated by comparing mild and severe RSV groups using Fisher’s exact text for categorical variables and Mann–Whitney test for continuous variables. *P* values < 0.05 are considered statistically significant.

^b^Tachypnea is defined as respiratory rate of ≥60 breaths/min in children <2 months, ≥50 breaths/min in children 2–11 months, and ≥40 breaths/min in children ≥12 months.

^c^Tachycardia is defined as heart rate of ≥180 beats/min in children <12 months and ≥140 beats/min in children ≥12 months.

Children in the severe RSV group had a higher median duration of hospitalization (6 vs. 4 days, *p* = 0.220), although the differences were not statistically significant. Children in the severe RSV group had a longer duration of oxygen administration (37 vs. 0 hours, *p* = 0.190). Oxygen was administered to children when the saturation level was less than 95% until the partial pressure of oxygen was raised. Oxygen flow rate and humidifier settings depend on the age of the child as per physician’s orders. Blood count results showed no significant differences in the counts of red blood cells (4.5 vs. 4.5 × 10^6^ cells/μL, *p* = 0.644), white blood cells (10.8 vs. 9.4 × 10^3^ cells/μL, *p* = 0.086), and platelets (32.5 vs. 33.3 × 10^4^/μL, *p* = 0.356) between children in the severe and mild RSV group. However, the severe RSV group had higher proportions of neutrophils (60.5% vs. 34.0%, *p* = 0.001), but lower proportions of lymphocytes (30.2% vs. 56.7%, *p* = 0.001) and monocytes (7.5% vs. 8.9%, *p* = 0.035) as compared with samples from the mild RSV group. Results from chest X-ray did not show any significant differences between groups. RSV subgroup A was the dominant subgroup in both RSV seasons and no associations were found between severity and RSV subgroup.

### Blood RNA expression profile

**Fig. 1:**
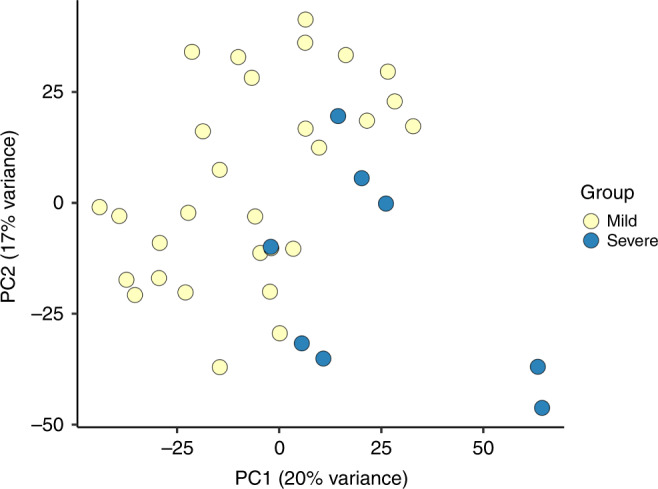
Global blood transcriptome profiling of children with mild and severe RSV infections. Principal component analysis plot of the 13,399 genes identified by RNA-sequencing showed that the first two principal components (PC1 and PC2) explain 20% and 17% of variance in gene expression between children in the severe RSV (blue circles) and mild RSV (yellow circles) group.

The median total RNA content per mL of whole blood was 4.8 μg (IQR 2.1–9.3 μg). About 500 ng of RNA was used during library preparation. The median RNA integrity number obtained was 8.0 (IQR 7.5–8.4). Sequencing depth of samples was averaged around 20 million reads with a mapping coverage of 86%. Expression data were available for 13,399 genes after filtering out genes with less than 10 reads as the sum over all samples. The global gene expression data were analyzed using principal component analysis (PCA) and results showed that the first principal component (PC1) explains for the 20% of the variance in the dataset with samples from the severe RSV group located on the lower right quadrant of the plot (Fig. 1). Clustering of samples by age and sex was not observed. However, samples from children by year of enrollment and hospital were mostly located at the bottom in the second principal component (PC2) and overlapped with samples from the mild RSV group (Supplementary Fig. S1). Gene loading of the top PC1 genes include CD177, IL1R2, MMP9, MMP8, and OLFM4 (Supplementary Fig. S2).

**Fig. 2:**
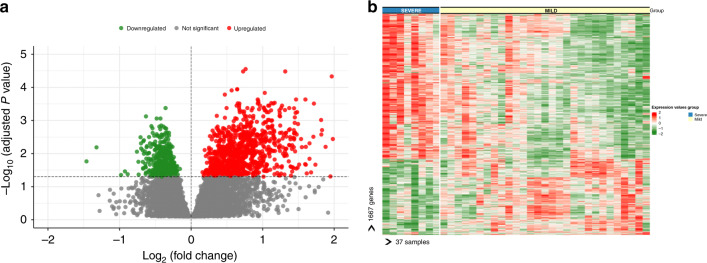
Gene expression profile in whole blood of children with mild and severe RSV infections. **a** Volcano plot of differentially expressed genes in children with RSV infection. The *X*-axis represents the log_2_ differences in gene expression between the two groups with higher positives values indicate increased gene expression (991 genes, red circles) while higher negative values indicate decreased gene expression (676 genes, green circles) in samples from children in the severe RSV group as compared with the mild RSV group. The *y*-axis represents adjusted *p* values (−log_10_ scale) for each gene with higher values indicate greater statistical significance, after adjusting for age, sex, and batch by enrollment year and hospital. The broken horizontal line indicates the 5% false discovery rate. **b** Heatmap of the 991 upregulated and 676 downregulated genes. Each row represents one gene and column represents one sample. Normalized expression is indicated as upregulated (red) or downregulated (green). Samples are grouped by severe RSV (blue) and mild RSV (yellow).

Differential gene expression analysis had identified 1667 genes with 991 upregulated and 676 downregulated genes in the blood samples of children from the severe RSV group as compared with children in the mild RSV group, after adjusting for year of enrollment, hospital, age, and sex at an FDR of 0.05 (Fig. 2a). The list of top upregulated genes included genes related to toll-like receptor (TLR1), while the top downregulated genes included genes related to transmembrane receptor tyrosine kinase signaling pathway (NOG and PDF) (Supplementary Table S1). Top loading PC1 genes, including CD177, MMP9, and MMP8 were upregulated in the RSV group. Heatmap analysis of the differentially expressed genes showed a distinct transcriptional expression pattern between children with severe and mild RSV (Fig. 2b).

### Transcriptional module analysis

**Fig. 3:**
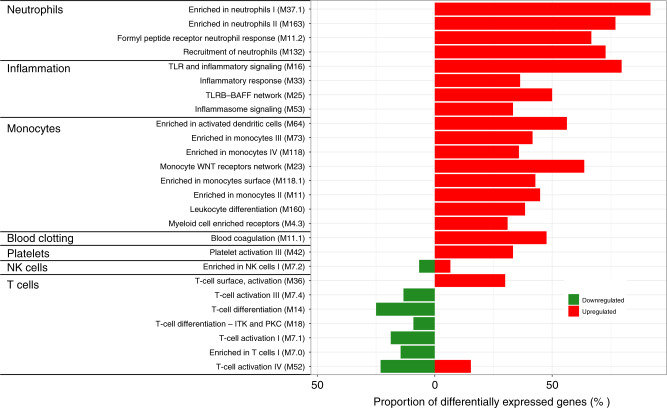
Transcriptional modular repertoire analysis in children with severe RSV. The difference between the proportion of upregulated and downregulated genes in each module when comparing children with severe and mild RSV are indicated in red and green bars, respectively. An increase in the proportion of upregulated genes was observed in modules related to neutrophil (M11.2, M37.1, M132, M163), inflammation (M16, M25, M33, M53), blood coagulation (M11.1), monocytes (M11, M23, M64, M73, M118, M118.1), and platelets (M42) in children in severe RSV. On the other hand, suppression of gene expression was observed in modules related to T cell activation (M7.1, M7.4, M54), and T cell differentiation (M14, M18).

Modular analysis of the differentially expressed genes showed that genes related to neutrophils had the highest proportion of upregulated genes (67–92%) in children in the severe RSV group when compared with children in the mild RSV group (Fig. 3 and Supplementary Table S2). Neutrophil modules with upregulated genes include enriched in neutrophils (M37.1 and M163), formyl peptide receptor neutrophil response (M11.2), and recruitment of neutrophils (M132).

The inflammation module is the second module containing high proportion of upregulated genes (33–80%). Identified inflammation modules include inflammasome signaling (M53), inflammatory response (M33), toll-like receptor (TLR) inflammatory signaling (M16), and TLR8 network (M25). Other modules with upregulated genes include monocytes (31–64%), blood coagulation (48%), and platelets (33%).

In contrast, most downregulated genes were identified in modules related to T cells (9–25%) in samples from the severe RSV group as compared with the mild RSV group. These T cells module include enriched in T cells (M7.0), T cell activation (M7.1, M7.4, M52), and T cell differentiation (M14 and M18).

### Gene network analysis

**Fig. 4:**
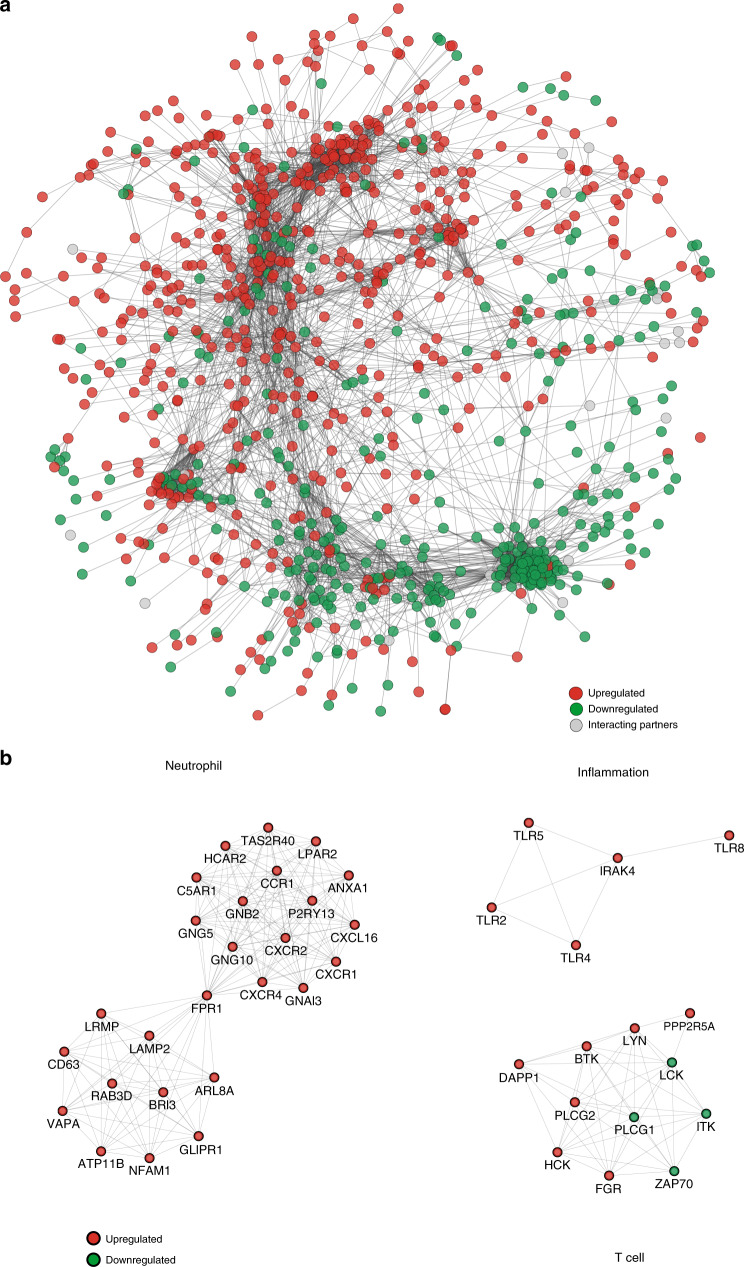
Gene co-expression network analysis in children with severe RSV. **a** Network analysis of differentially expressed genes identified between severe and mild RSV groups. The network consists of 978 nodes and 6517 interactions. Red and green circles represent upregulated and downregulated genes, respectively. Gray circles represent interaction partners. **b** Representative subnetwork hubs with highly interconnected nodes.

The generated gene co-expression network has 978 genes and 6517 interactions (Fig. 4a). Genes are connected in the network if a protein–protein interaction is identified in the STRING database. The network topology showed one large component and the rest of the genes that are not connected were not included in the network. The differentially expressed genes that belonged to the large component were organized into co-expression network hubs as identified by MCODE (Fig. 4b and Supplementary Fig. S3). Of the differentially expressed genes, 86 belonged to network hubs and gene modules. Of these, 9 genes code for G-protein-coupled receptors (C5AR1, CCR1, CXCR1, CXCR2, FFAR2, FPR1, FPR2, P2RY13, PTAFR), 11 genes code for kinases (BTK, FGR, HCK, IRAK3, ITK, LCK, LYN, PAK1, PRKCD, PRKCQ, ZAP70), and 6 genes code for toll-like receptors (TLR1, TLR2, TLR4, LR5, TLR6, TLR8).

### Drug–target network analysis

**Fig. 5:**
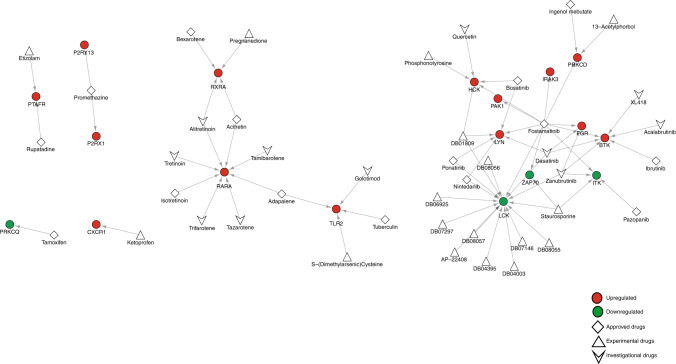
Drug–target network analysis in children with severe RSV. The network consists of 43 drugs and 18 drug targets. Red and green circles represent drug targets that are upregulated and downregulated in this study. Diamonds represent FDA-approved drugs, triangles represent experimental, and arrowheads represent investigational drugs.

The generated drug–target network is composed of 43 drugs and 18 genes that code for proteins as drug targets with 69 interactions (Fig. 5). The network has six components and the topology is dominated by a single component that is highly connected due to the drug, Fostamatinib, which interacts with 10 drug targets (BTK, FGR, HCK, IRAK3, ITK, LCK, LYN, PAK1, PRKCD, ZAP70). Drug targets that interact with several drugs also contributed to the connectivity of the network, such as LCK, which is targeted by 16 drugs. Of the 43 drugs in the network, 15 are approved by US FDA, 11 are investigational, and 17 are experimental drugs.

## Discussion

Analysis of the gene expression profiles of whole blood in children infected RSV revealed an imbalance in innate and adaptive immune responses. We observed that children with severe RSV had upregulated genes involved in neutrophil recruitment and activation, inflammatory response, monocyte, blood clotting, and platelets when compared with children having mild RSV infection. In contrast, we observed downregulation of genes involved in T cell activation and differentiation in children with severe RSV.

Modular analysis showed that genes related to neutrophils were associated with severe RSV infection. Neutrophils were the major white blood cell population in the upper and lower respiratory tracts of children with RSV infection.^33,34^ Neutrophils from the blood migrate to the lungs as a response to chemokines released at the site of infection, which results in the destruction of infected cells via the release of neutrophil-derived proteases.^35^ However, these proteases released by neutrophils also damages surrounding healthy cells, which may result in lung injury.^35^ In this study, we observed the upregulation of matrix metallopeptidase (MMP8, MMP9) genes, which were associated with neutrophil activation and migration and the overexpression of these genes in children infected with RSV having severe lower respiratory tract infection is in agreement with previous studies,^6,7,36–38^ We also observed in this study the upregulation of the formyl peptide receptor genes (FPR1 and FPR2), which were identified in the neutrophil module and network hub, suggesting its central role in the neutrophil response pathway. This observation will require further investigation with regard to the molecular mechanism of FPR1 and FPR2 in the pathogenesis of severe RSV. These findings suggest an important link between neutrophils and severe RSV infection and provide a potential marker of RSV disease severity.

Genes related to inflammatory response were upregulated in children with severe RSV. Overexpression of genes related to inflammatory response was also observed previous studies.^6,39^ In this study, we identified overexpression of toll-like receptor genes (TLR1, TLR2, TLR6) that are associated with inflammatory response in children with severe RSV. In a mouse hypoxia model, expression of TLR2 and TLR6 genes was associated with hypoxia in addition to their innate immune response.^40^ In the present study, children in the severe RSV group have lower peripheral oxygen saturation levels than children in the mild RSV group. These results suggest the importance of hypoxia-elicited signaling pathways in inflammatory immune response in increasing our understanding of the pathogenesis of RSV. To complement SpO_2_ measurement, TLR2 and TLR6 can be explored as potential molecular markers of severe RSV infection.

Gene modules related to blood coagulation, monocytes, and platelets were upregulated in children with severe RSV. During infection, blood coagulation is activated to limit the spread the of the pathogen; however, overactivation may occur resulting to thrombosis, excessive inflammation, and tissue damage.^41^ In this study, we observed in children with severe RSV had an overexpression of a tyrosine-protein kinase LYN gene, which is associated with blood coagulation and platelet signaling.^42^ Another upregulated gene we observed is the G-protein-coupled platelet-activating factor receptor (PTAFR) gene. In vitro studies, PTAFR expression is induced upon rhinovirus infection^43^ and serves as an attachment protein of *Streptococcus pneumoniae.*^44^ Drug–target analysis identified two receptor antagonists (rupatadine, etizolam) to PTAFR and five kinase inhibitors to LYN. Children in the severe RSV group did not show any clinical manifestations of coagulopathy and laboratory tests for disrupted blood clotting were not performed in this study. It remains to be seen whether LYN and PTAFR are associated with coagulation in severe RSV.

Gene modules related T cell response were mostly downregulated in children with severe RSV. This finding is in line with previously reported transcriptomic studies.^6^^[,45^ Poor T cell response including low cell count and low lymphoproliferative response was observed in children with severe RSV who required mechanical ventilation.^46^^[,47^ Low levels of T cell cytokines (interleukin 2 (IL2), IL4, interferon-γ, IL17) were observed in children with severe RSV, suggesting the suppression of adaptive cytotoxic T cell lymphocyte responses and natural killer (NK) cells and the reliance of innate immune responses mediated by neutrophils in neutralizing the virus.^48^ In this study, we observed the downregulation of CD8A and CD8B genes, which are cytotoxic T cell markers and ZAP70 (zeta chain of T cell receptor-associated protein kinase 70) gene, which is related to Th1- and Th2-lymphocyte response. This suppression of gene expression may shift the balance towards Th2 response that is associated with enhanced RSV pathogenicity.^49^ In a murine in vitro study, ZAP70 kinase activity is high in resting Th2 cells, and the activity decreased in activated Th2 cells;^50^ however, the molecular mechanism of ZAP70 and its role in the development of severe RSV disease are unknown. Drug–target network analysis identified staurosporine, an experimental drug and fostamatinib, an FDA-approved drug as inhibitors of ZAP70. Since ZAP70 is a kinase and it is downregulated in children with severe RSV, then these drugs are not suitable medication but drugs that could stimulate the cytotoxic T cell response pathway could be a promising option in the treatment of children with severe RSV.^51^

This study has several limitations. Our transcriptome data provided a snapshot of gene expression at a single time point upon enrollment to the study. Thus, we cannot assess the genes that are responsible for severe RSV, which can be addressed by a longitudinal study design. This study has a small sample size and lacked statistical power to make strong statements on the associations between gene expression and severe RSV. Nonetheless, our results are in agreement with previous reports that have larger sample size,^6,45^. Another limitation of this study is that we did not perform cell subset analysis (e.g. CD4+, CD8+ lymphocytes, etc.) since this was not routinely performed in the hospital study sites. In the analysis, we did not adjust for cell count in the blood sample since we did not observe significant differences between the two groups. An advantage of this study is that we used the WHO definition for severe RSV, which is based objective parameters such as SpO_2_ measurements and easy to implement in various hospital settings.

## Conclusions

We observed overexpression of genes related to neutrophil and inflammatory response and under expression of genes related to T cell response in children with severe RSV. By combining transcriptome profiling and network analysis, we were able to identify genes that are associated with disease severity and drugs that can be repurposed as potential treatment options of severe RSV.

## Supplementary information

Supplementary Figure S1-S3

Supplementary Table S1

Supplementary Table S2

## Data Availability

The RNA-seq data used in this study have been deposited in NCBI Gene Expression Omnibus database with accession number GSE155925. Codes and data used for the RNA-seq analysis are available in GitHub at [https://github.com/clyde-dapat/transcriptome-analysis-severe-RSV].
