# Supplementary material for: Gene signature of children with severe respiratory syncytial virus infection
Source: Pediatr Res. 2021 Jan 28;89(7):1664–72. doi: 10.1038/s41390-020-01347-9 (PMC8249238; doi:10.1038/s41390-020-01347-9)

Supplementary Figure S1. PCA by WHO LRTI

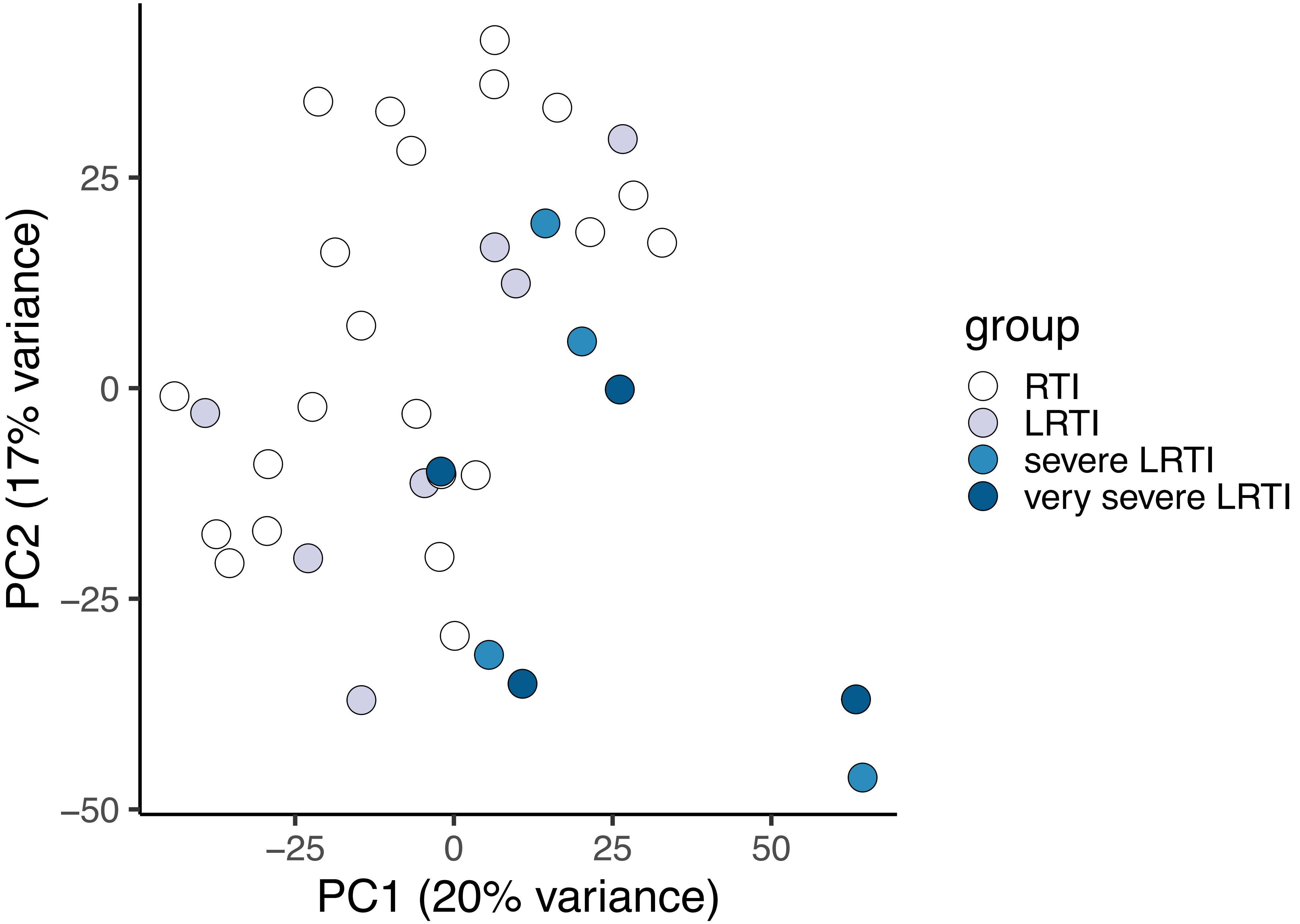

Supplementary Figure S1. PCA by year of enrollment

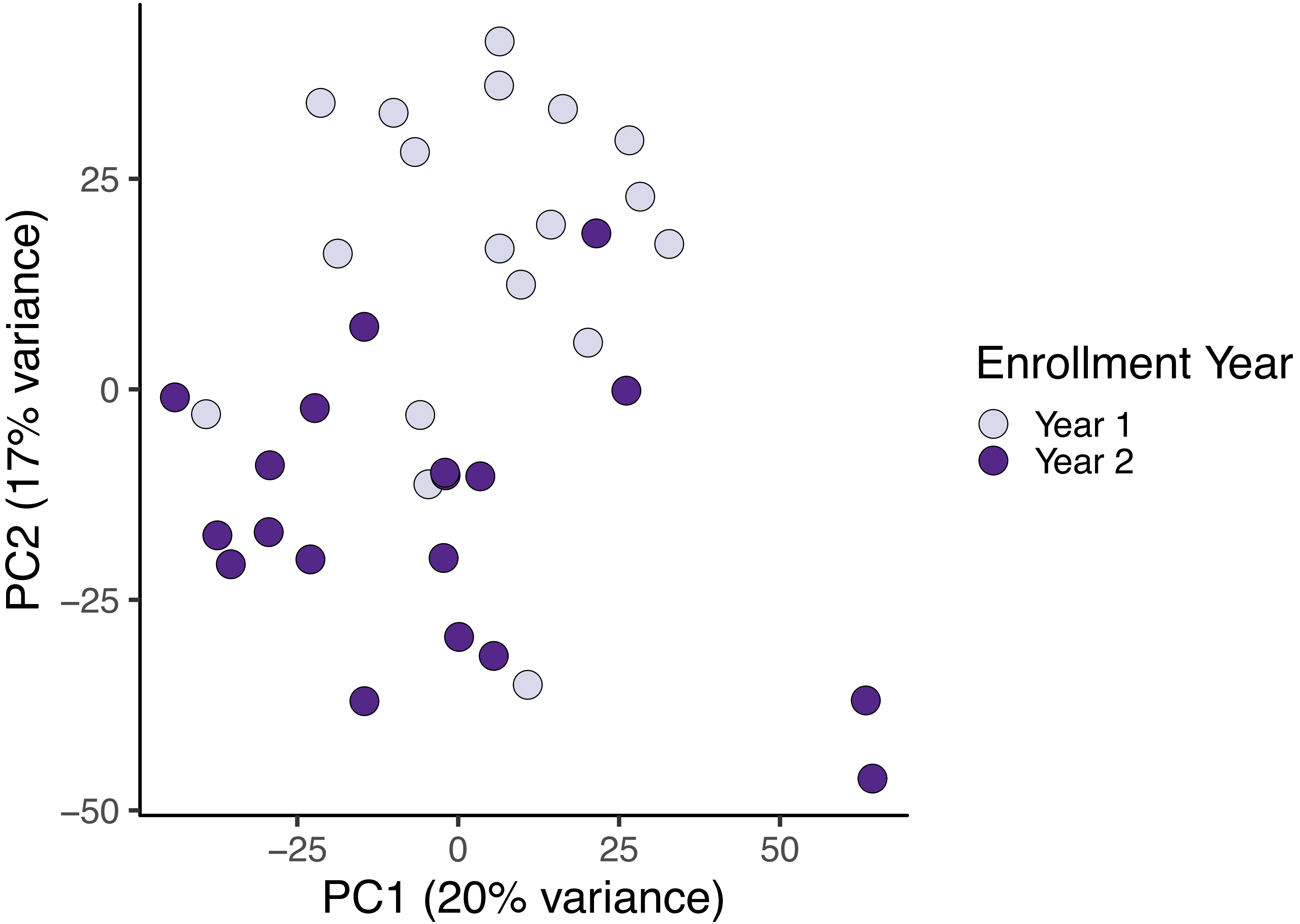

Supplementary Figure S1. PCA by hospital

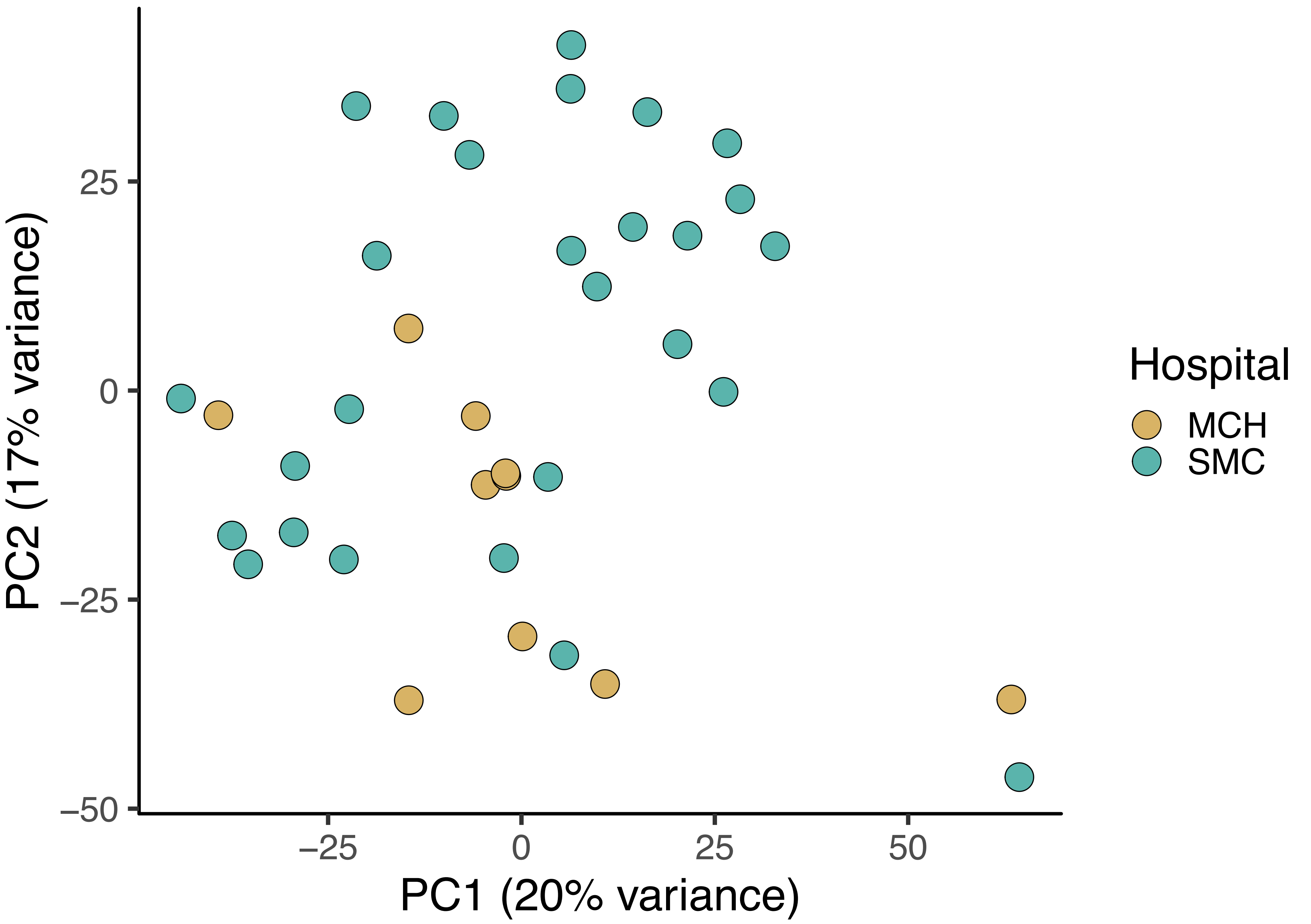

Supplementary Figure S1. PCA by sex

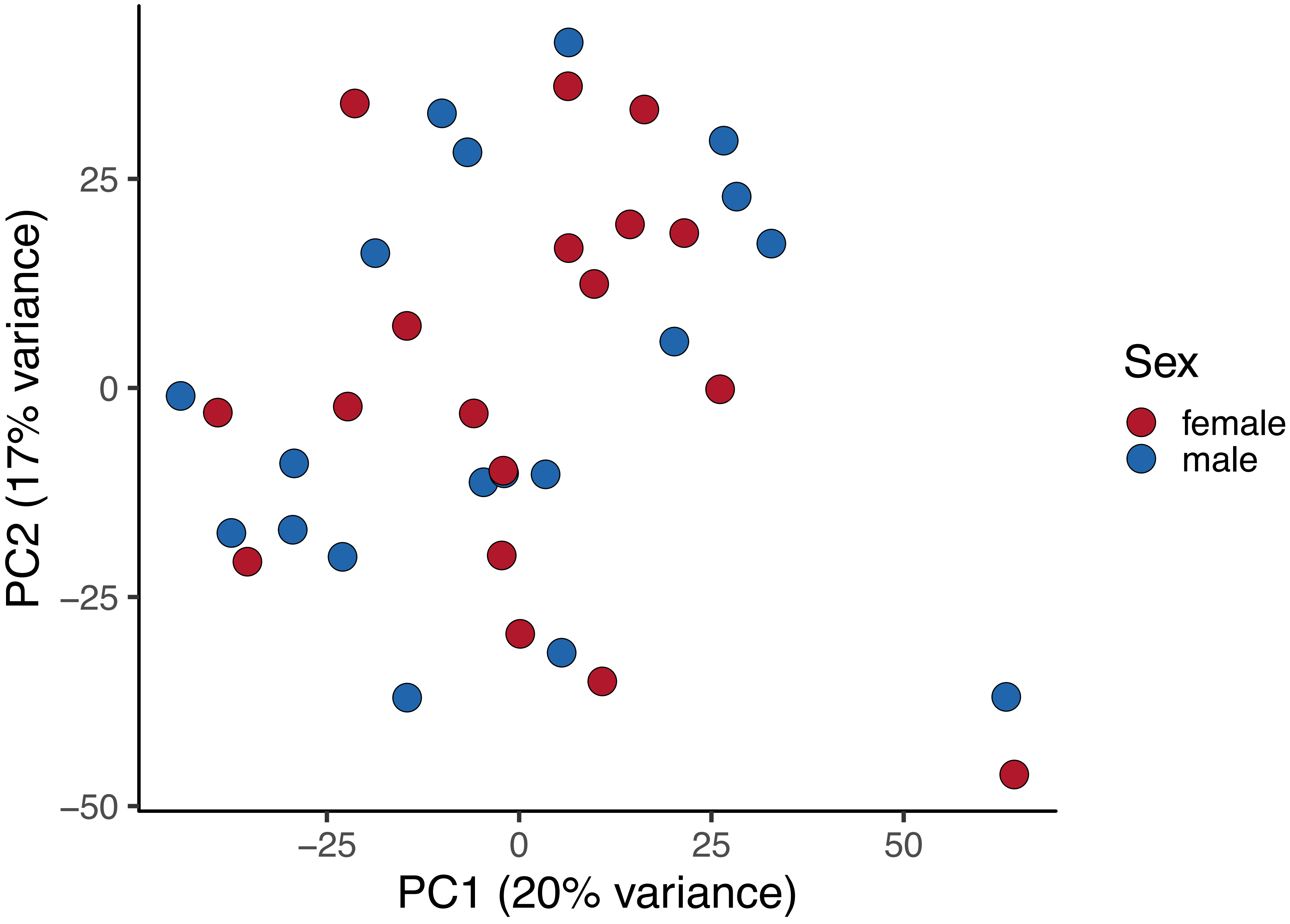

Supplementary Figure S1. PCA by age

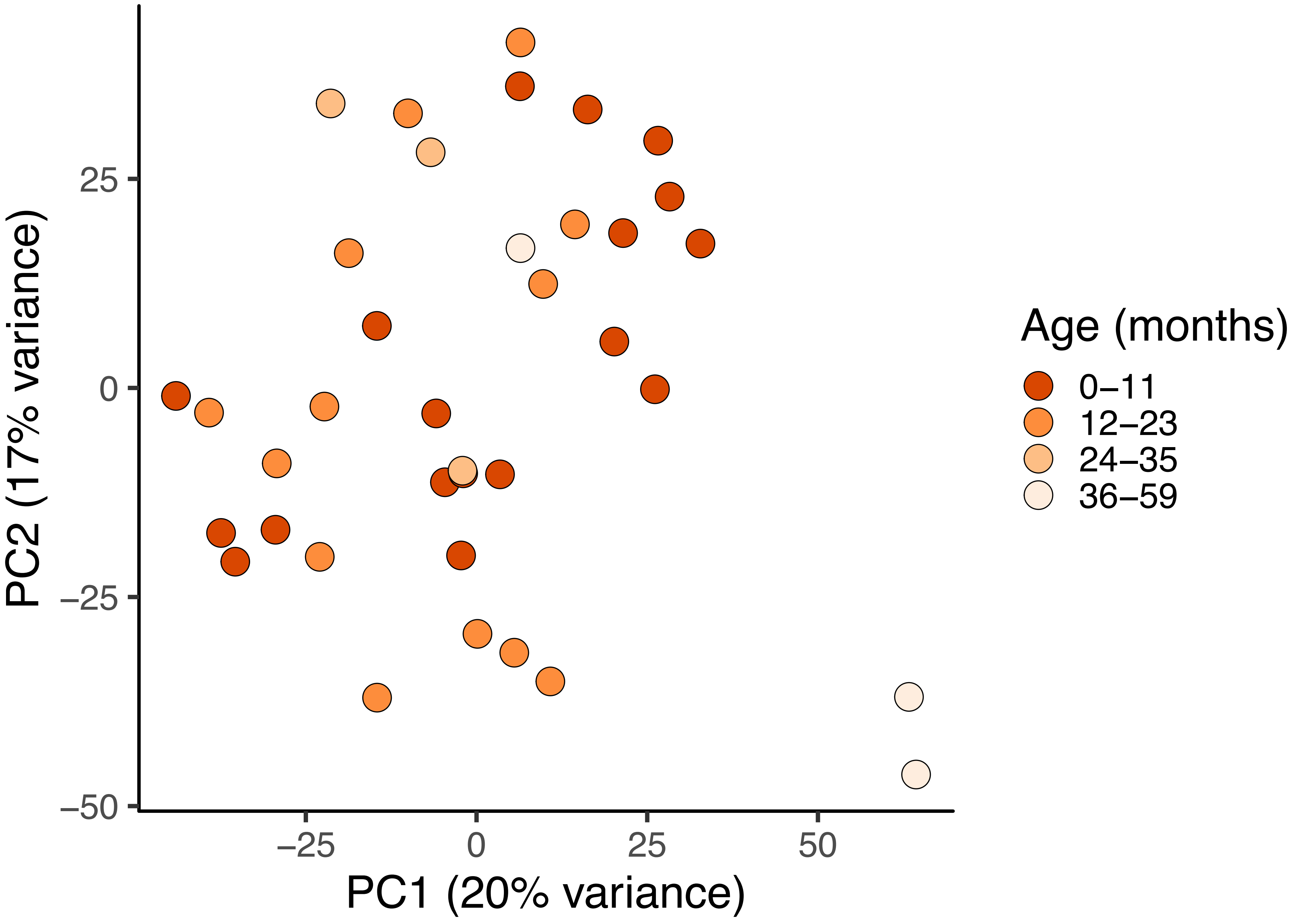

Supplementary Figure S2. Top and bottom PC loadings

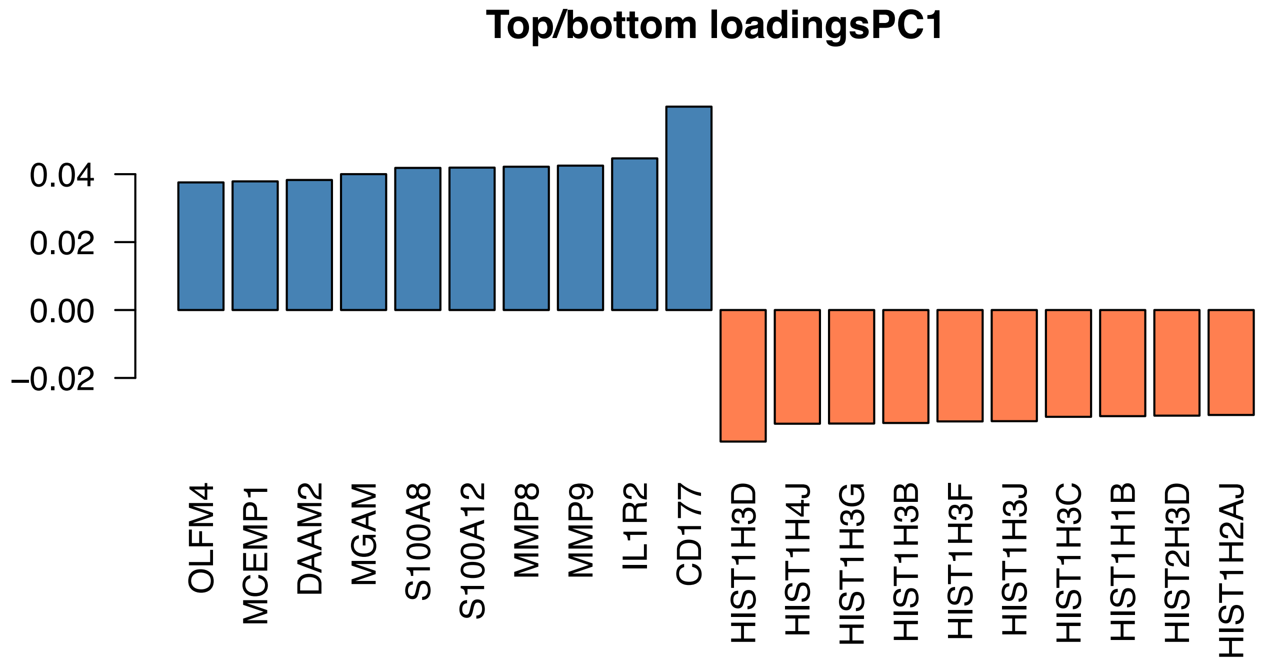

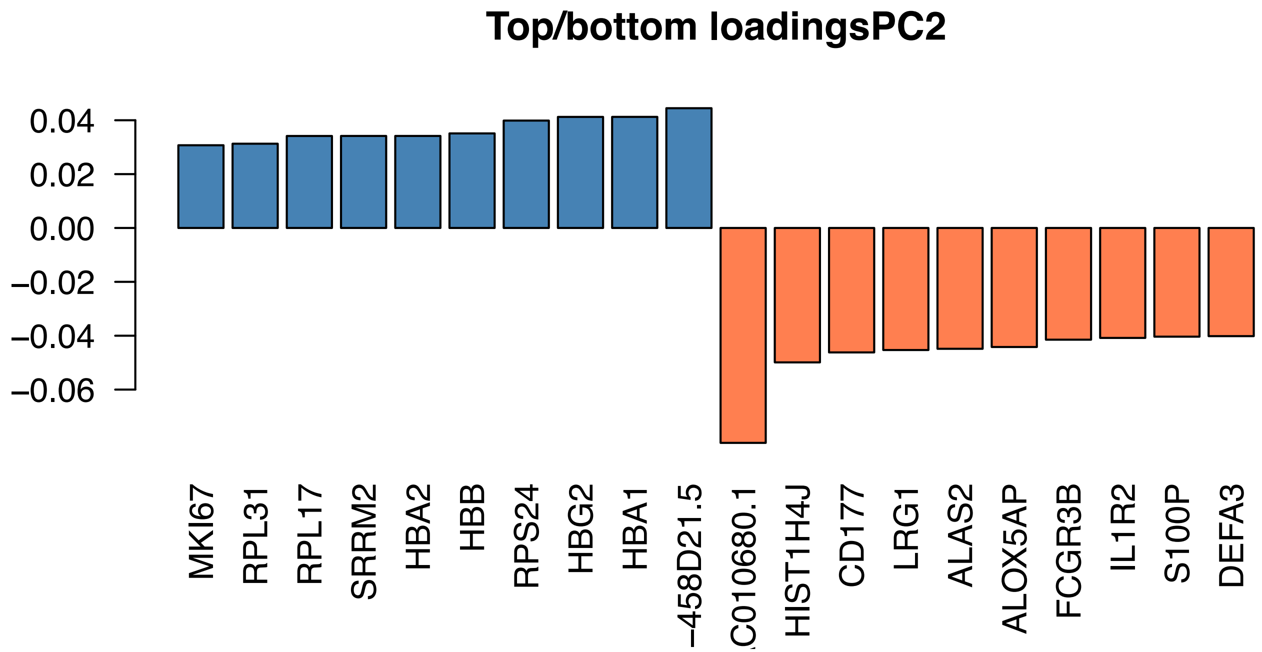

Supplementary Figure S3. MCODE analysis

A. rRNA processing

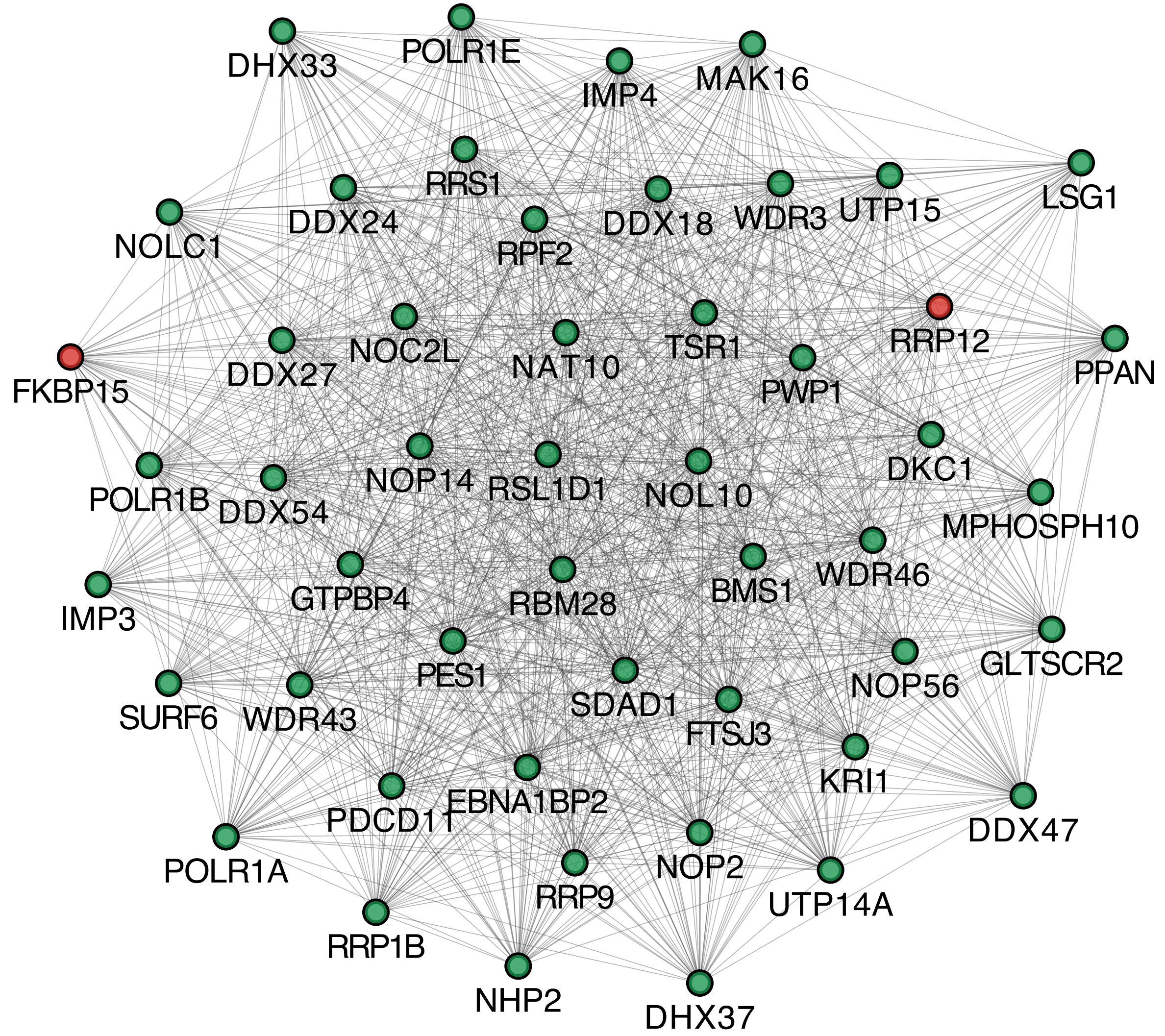

Supplementary Figure S3. MCODE analysis

B. protein ubiquitination

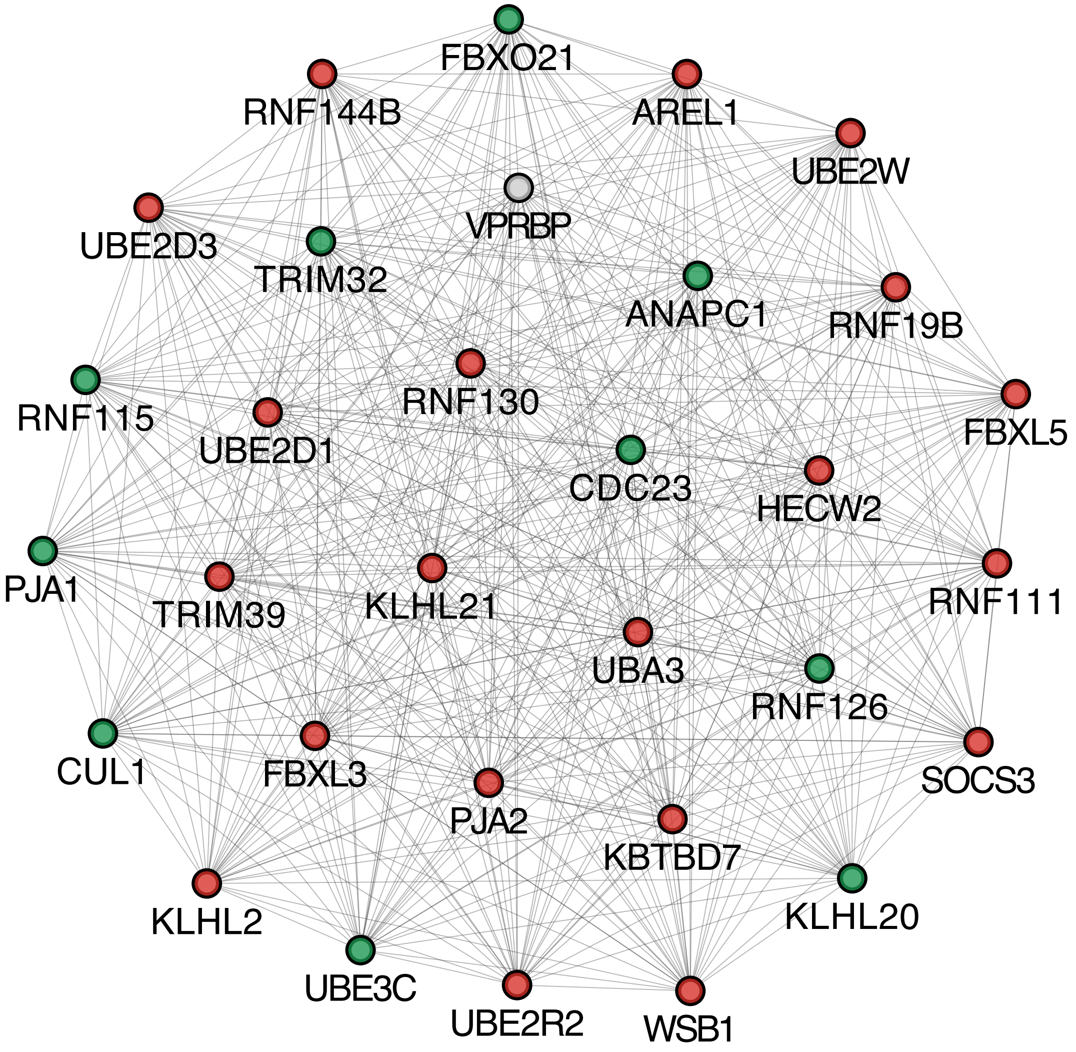

Supplementary Figure S3. MCODE analysis

C. blood coagulation

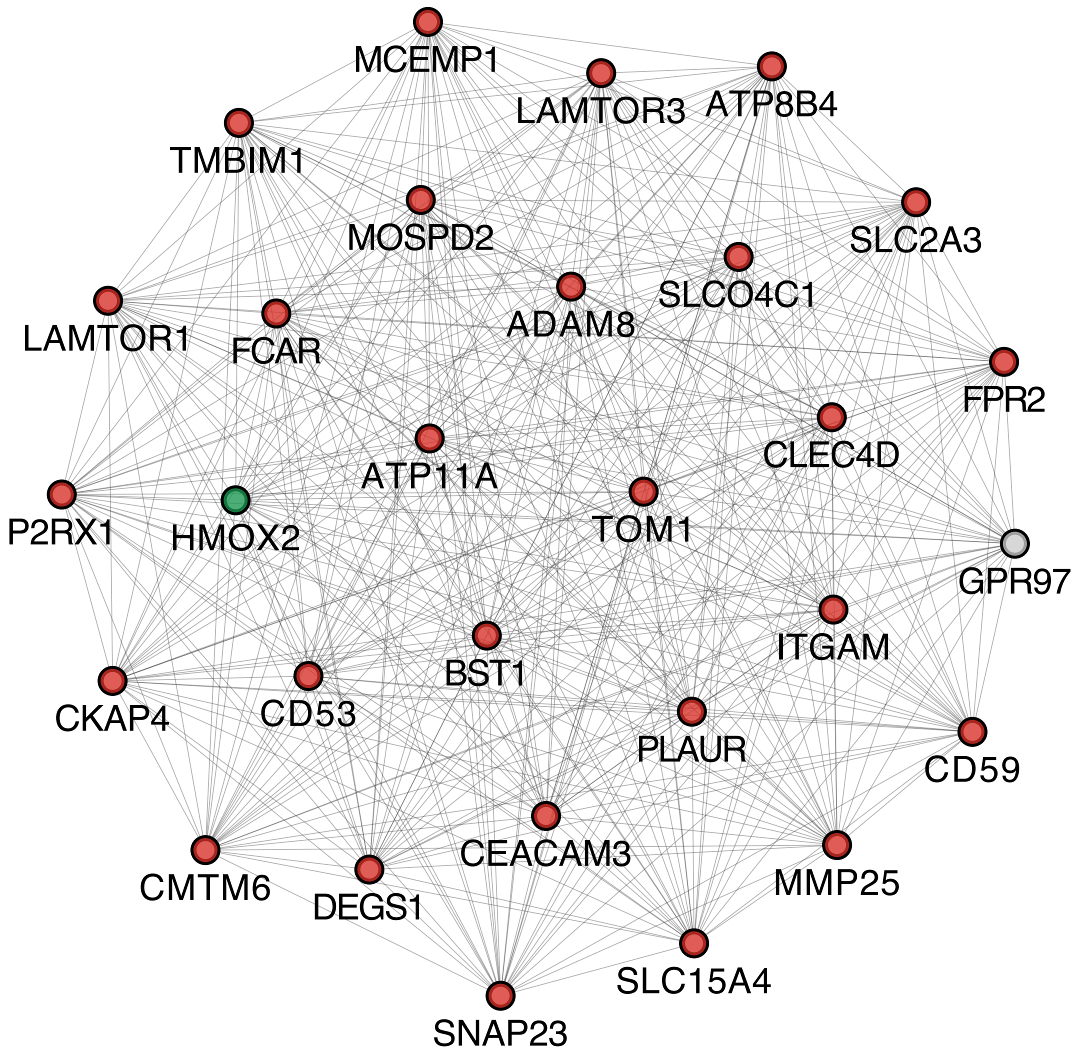

Supplementary Figure S3. MCODE analysis

D. RNA splicing

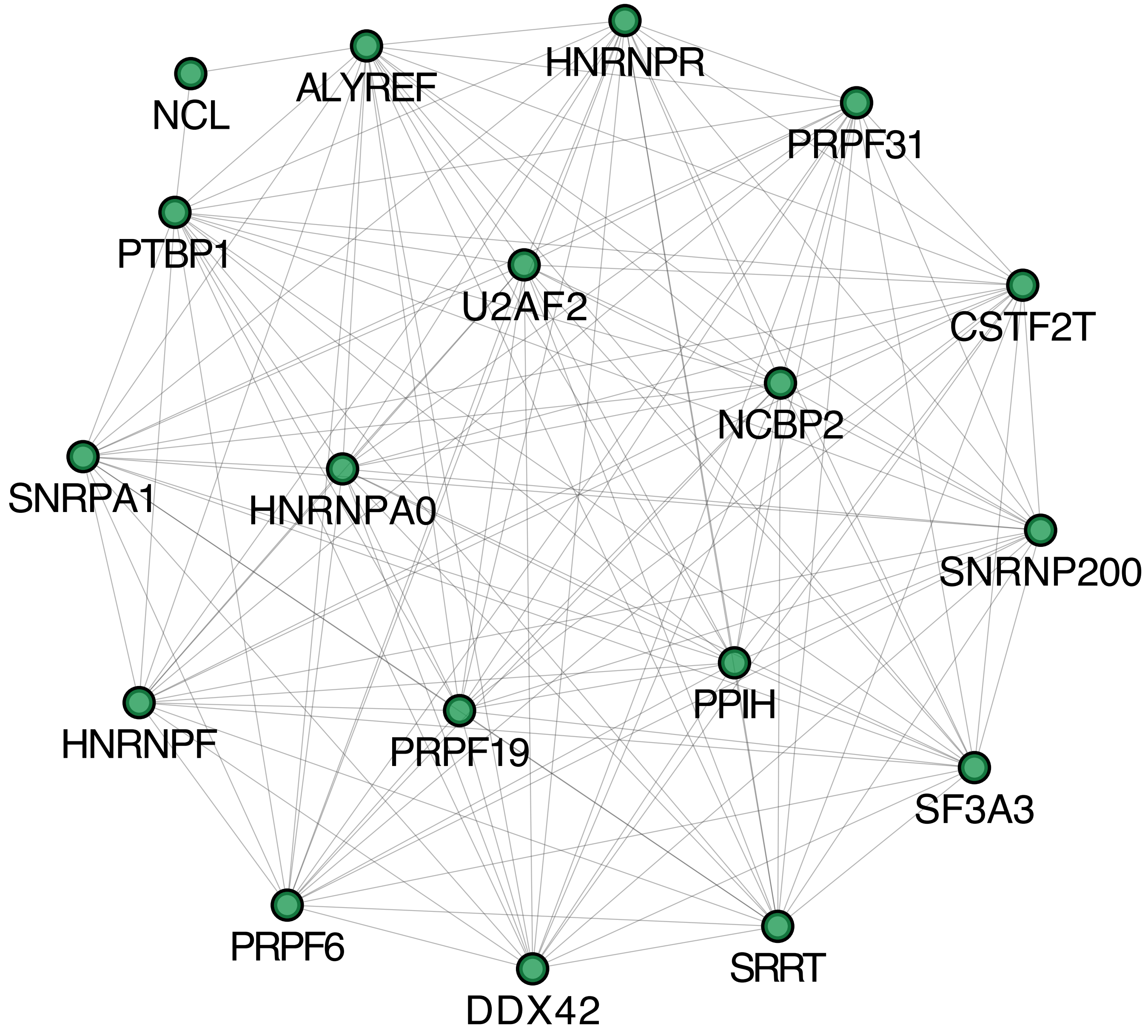

Supplementary Figure S3. MCODE analysis

E. chemotaxis

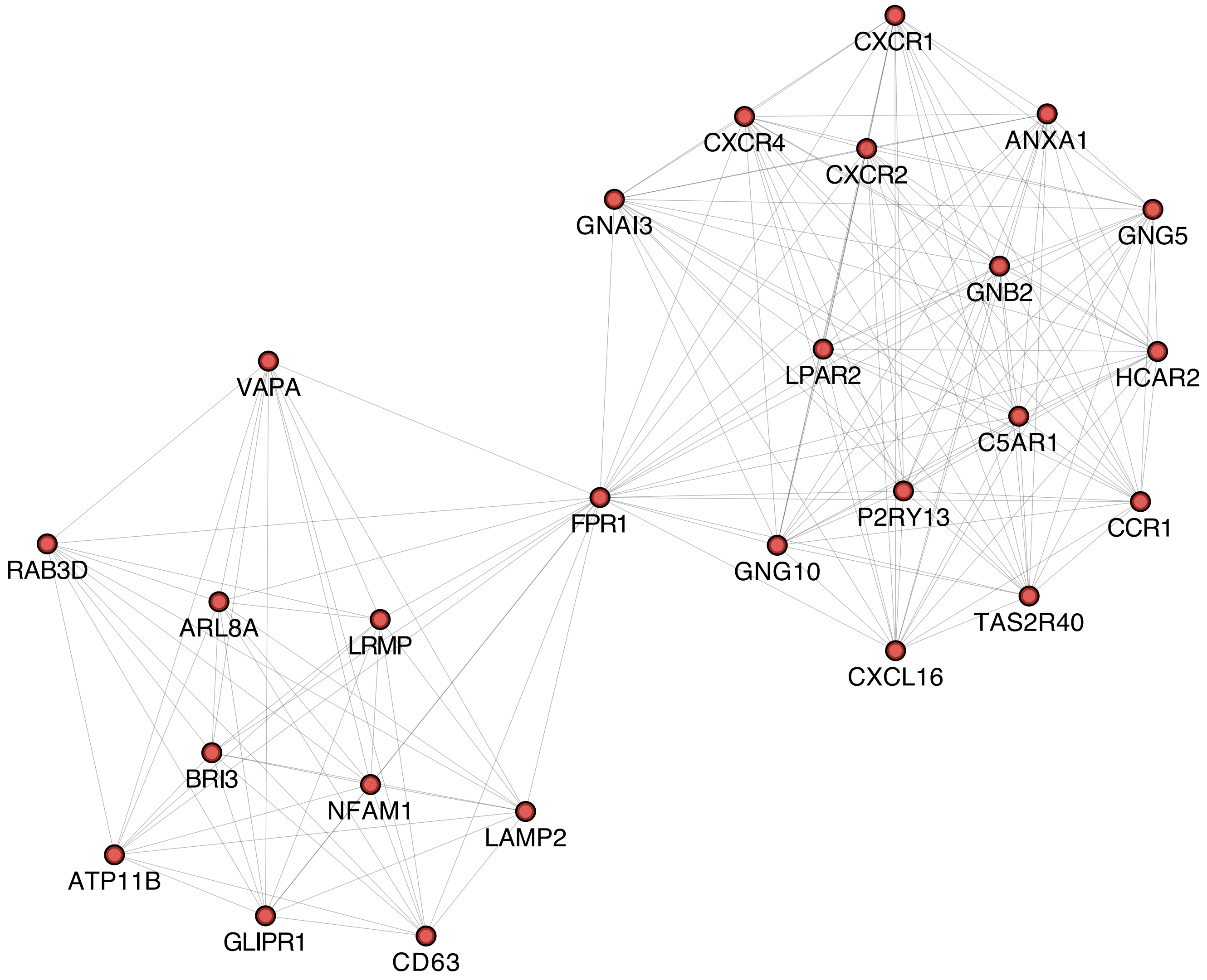

Supplementary Figure S3. MCODE analysis

F. cytokine production

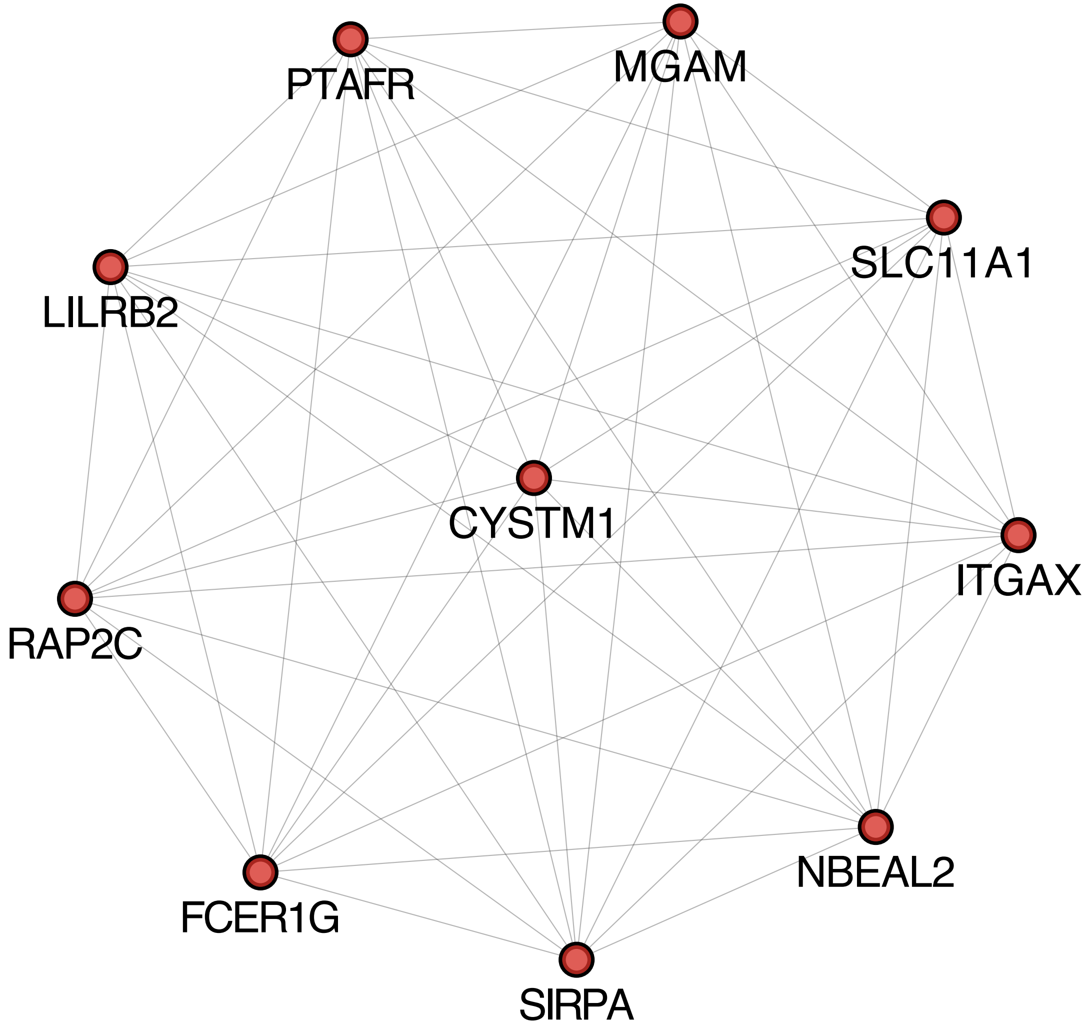

Supplementary Figure S3. MCODE analysis

G. lymphocyte proliferation

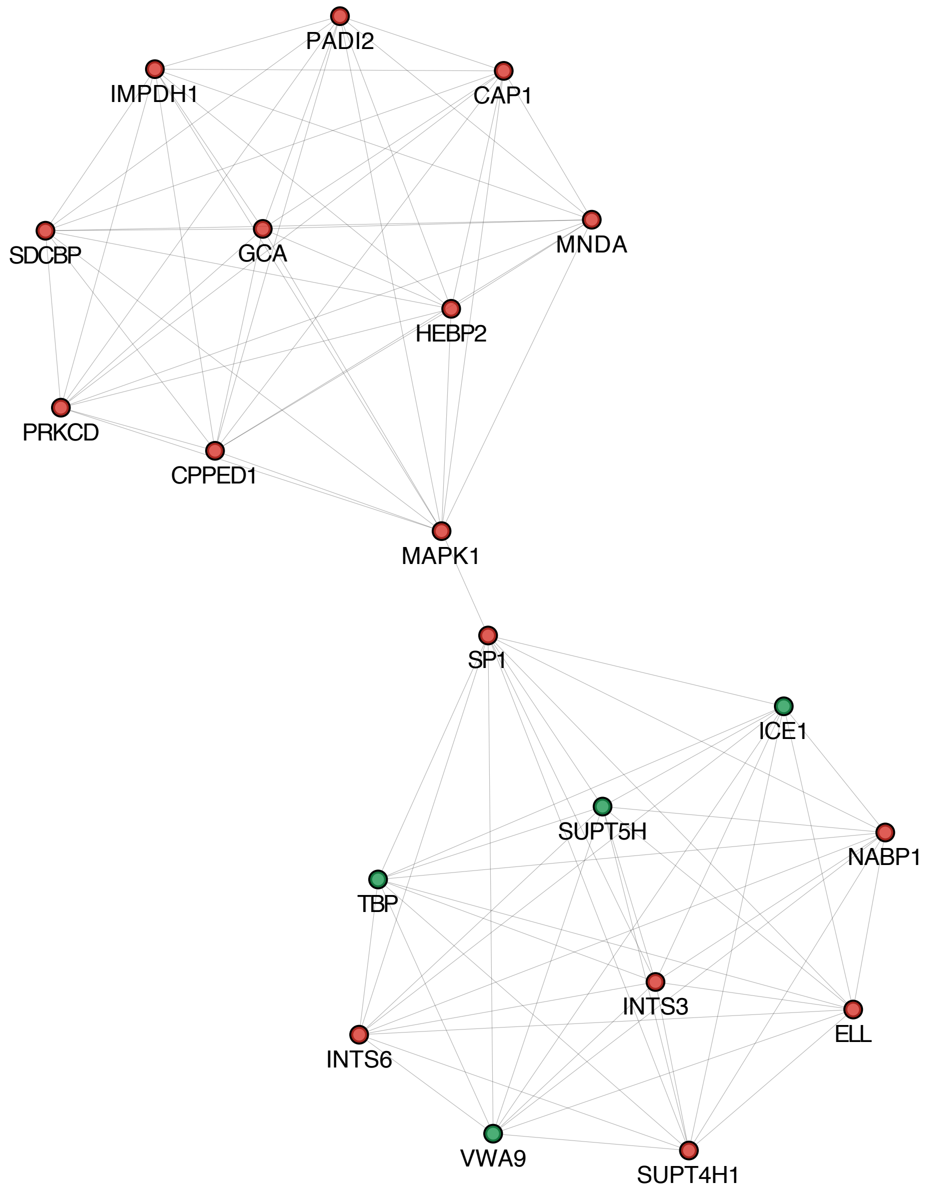

Supplementary Figure S3. MCODE analysis

H. T cell receptor signaling

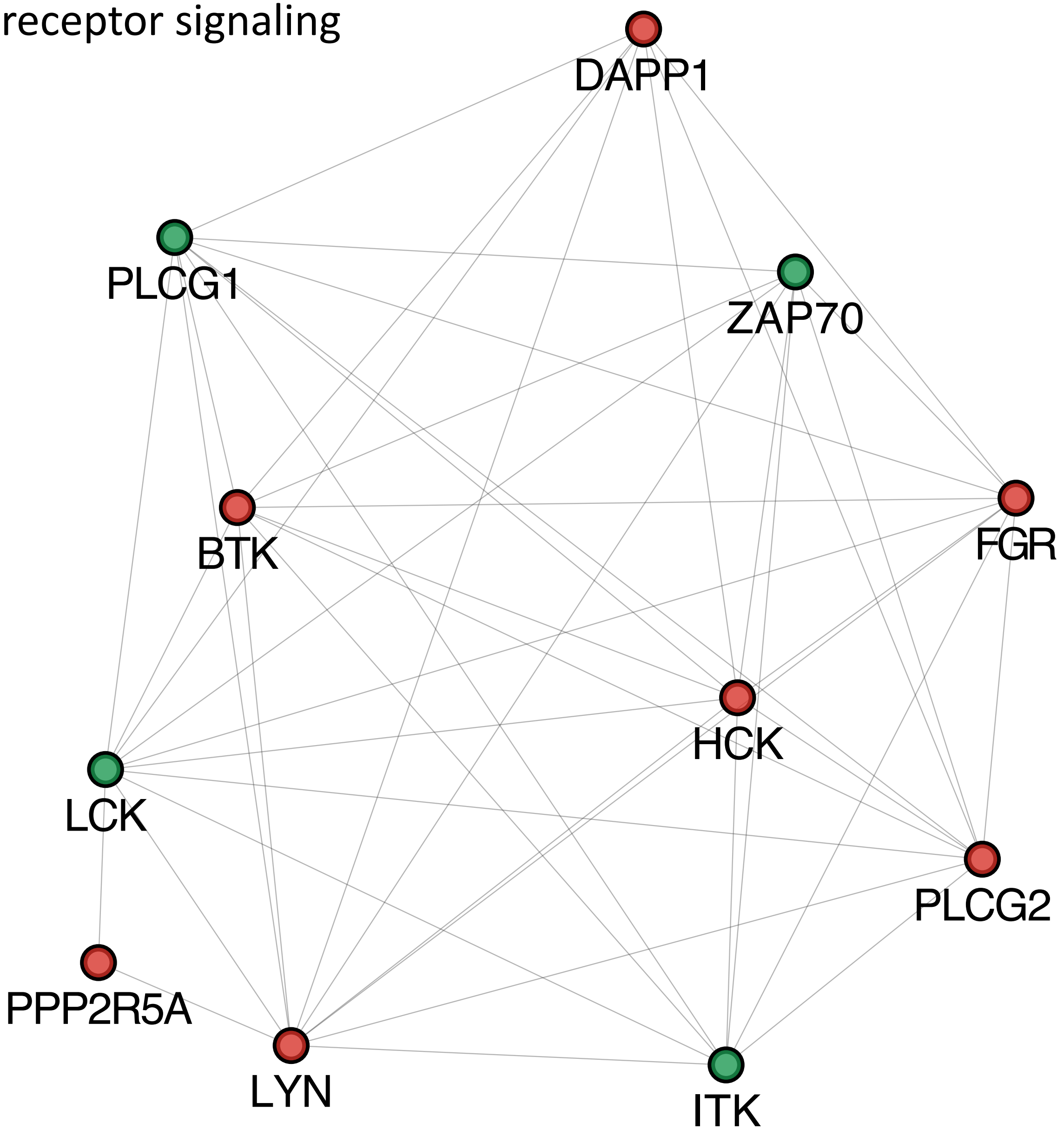

Supplementary Figure S3. MCODE analysis

I. cell cycle

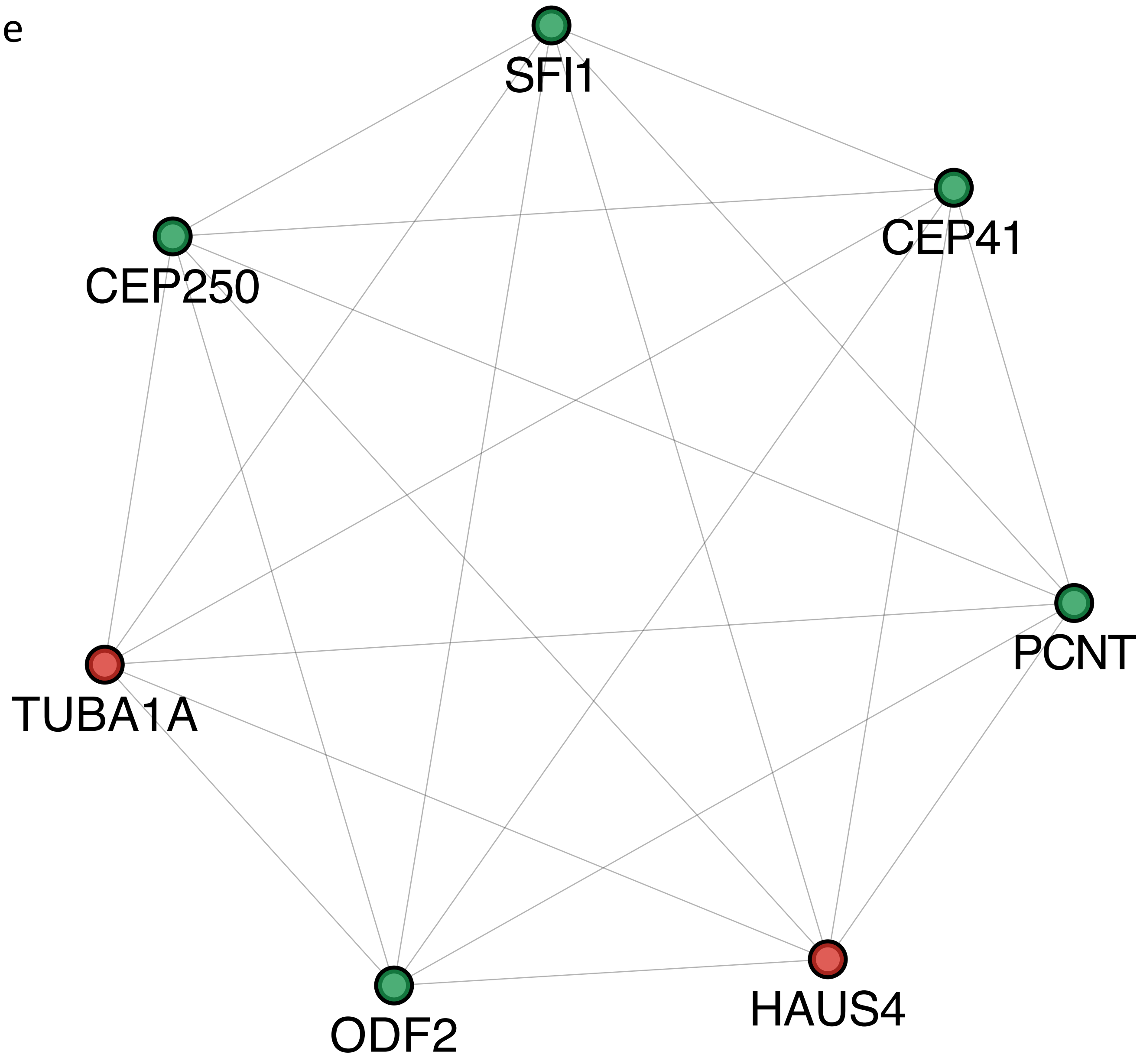

Supplementary Figure S3. MCODE analysis

J. glycolysis

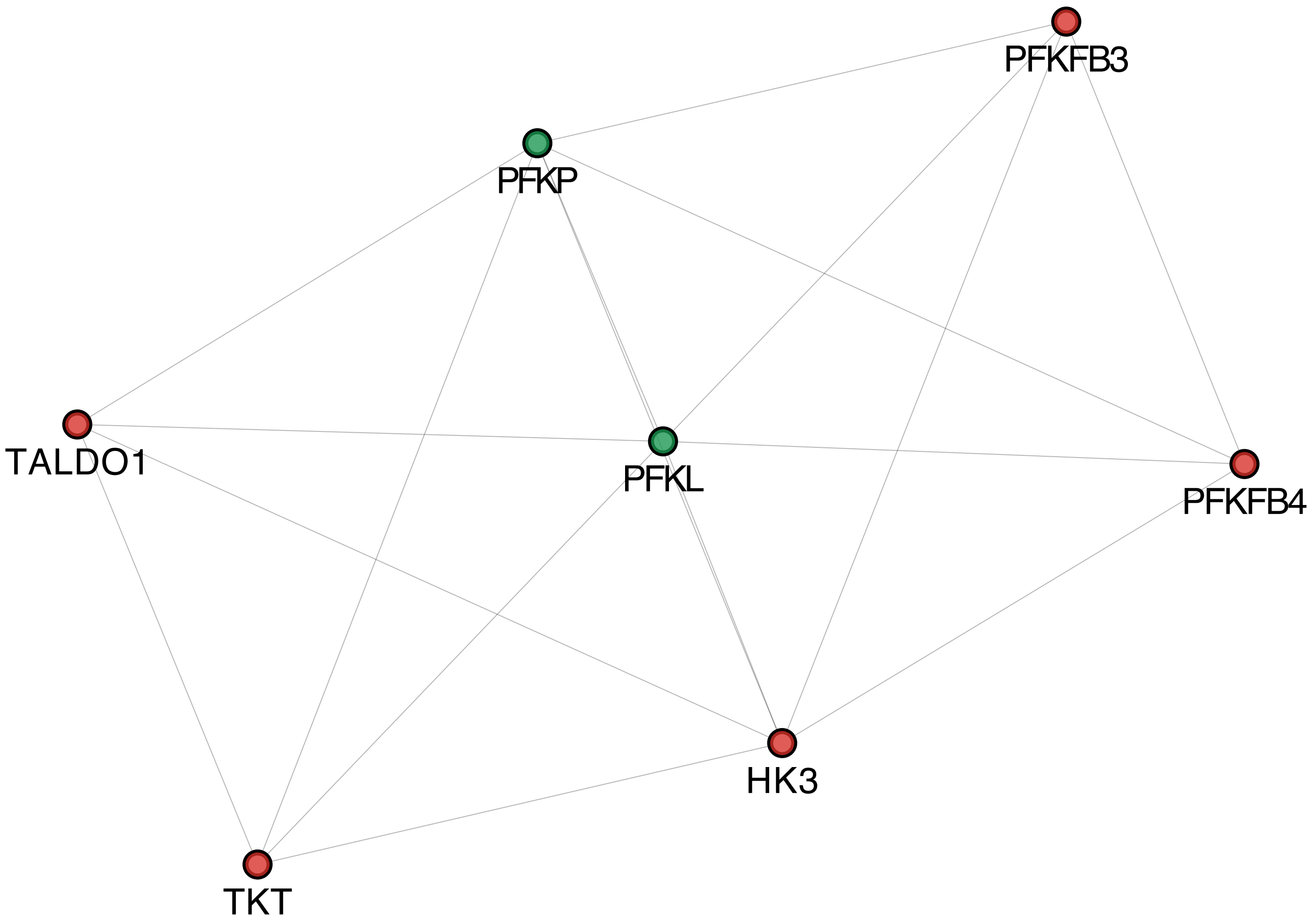

Supplementary Figure S3. MCODE analysis

K. G protein-coupled receptor signaling

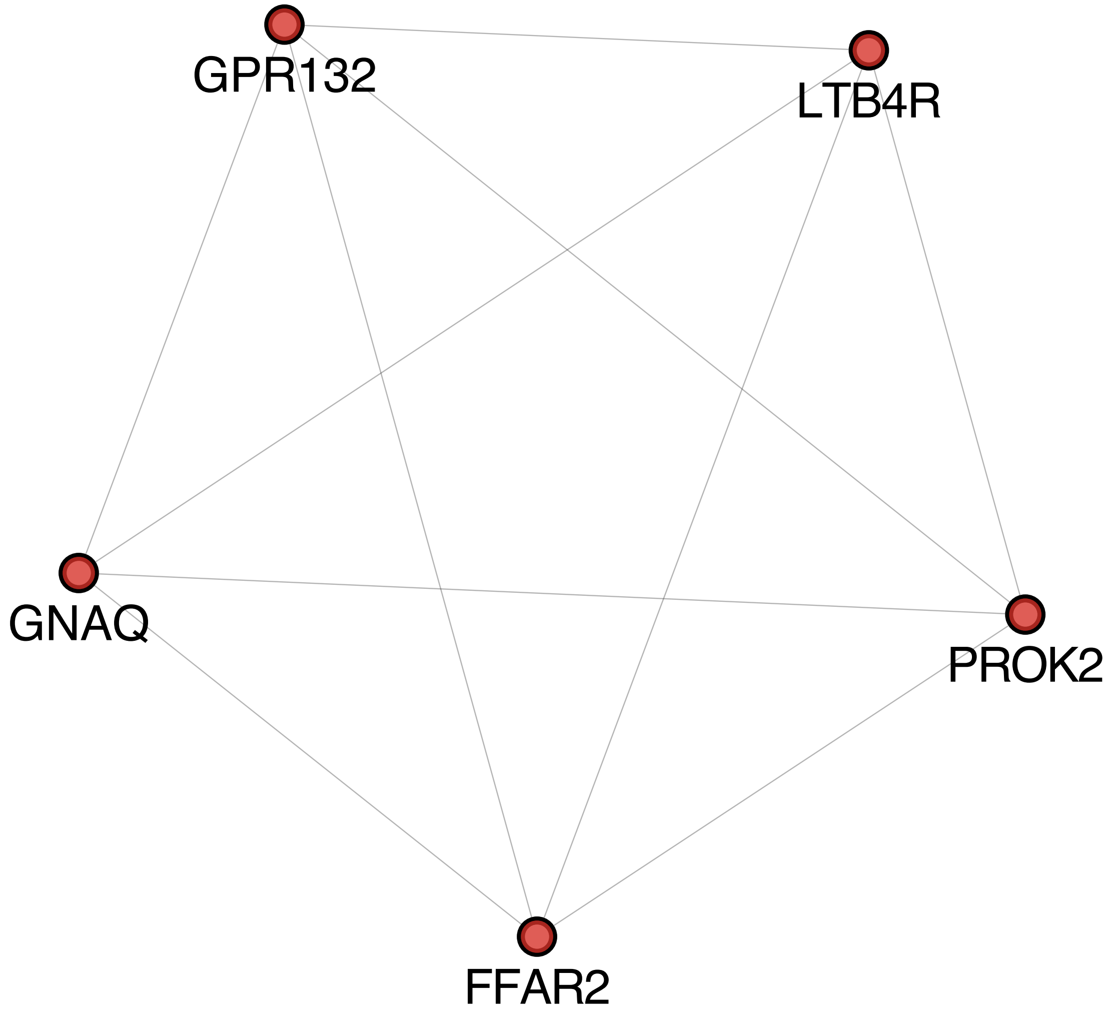

Supplementary Figure S3. MCODE analysis

L. T-helper 2 (Th2) cell differentiation

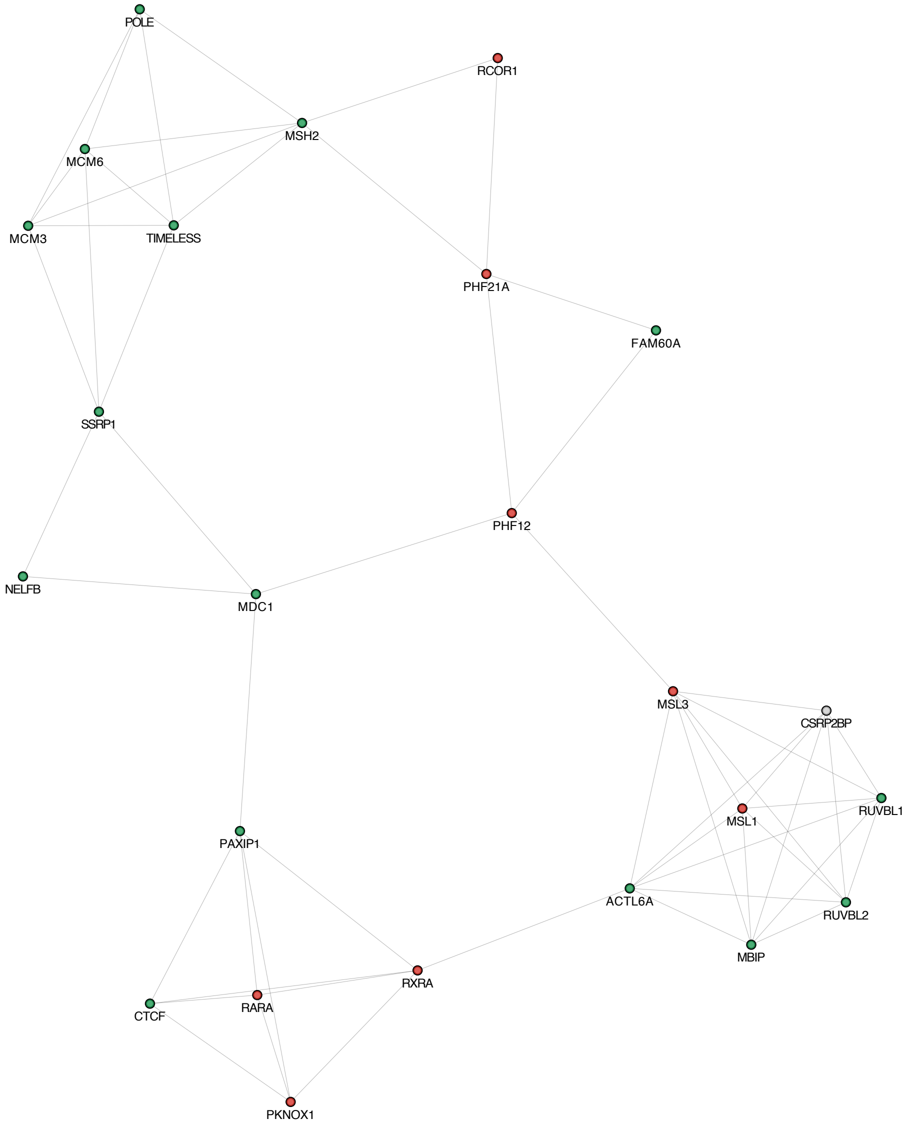

Supplementary Figure S3. MCODE analysis

M. ATP synthesis coupled proton transport

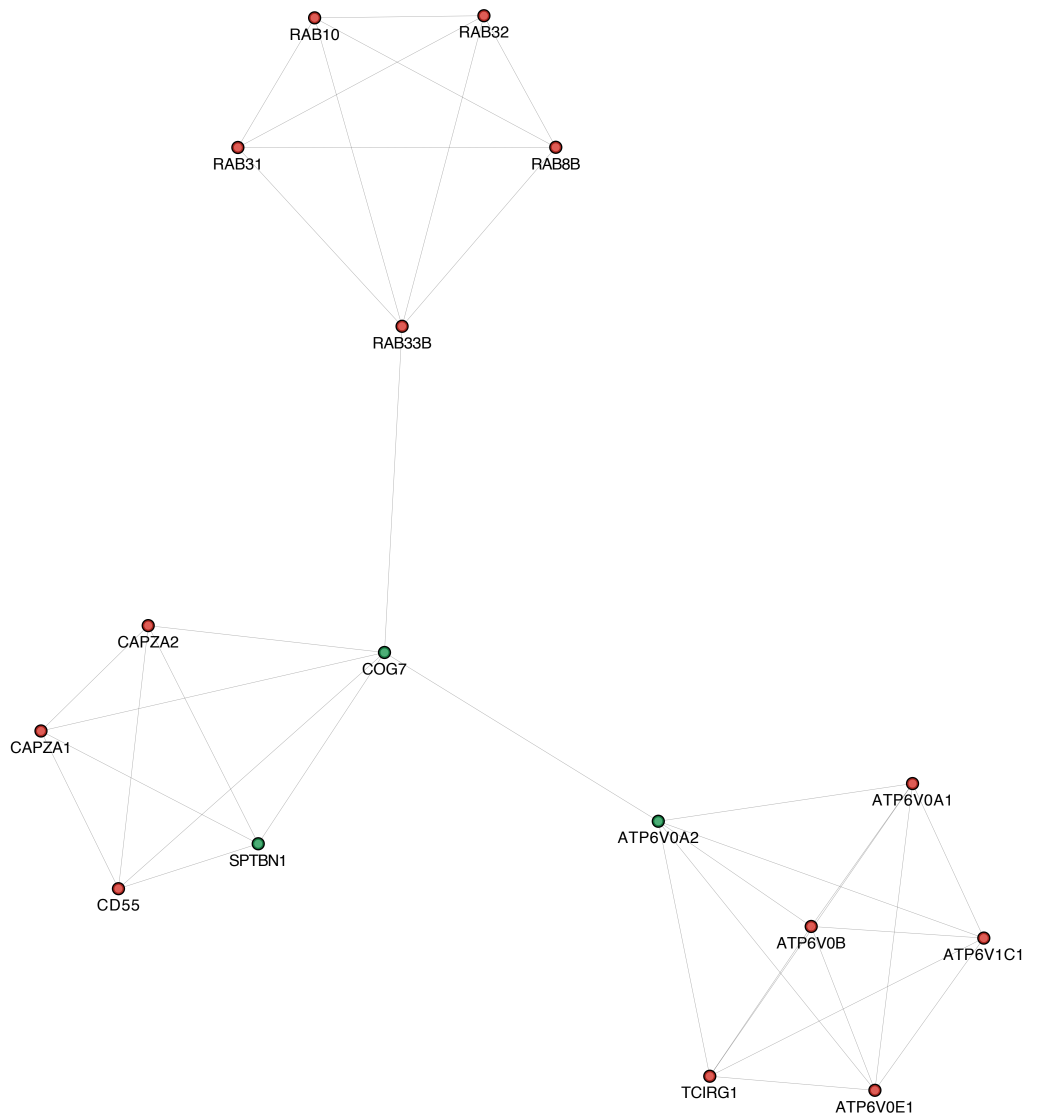

Supplementary Figure S3. MCODE analysis

N. Regulation of IL-2 production

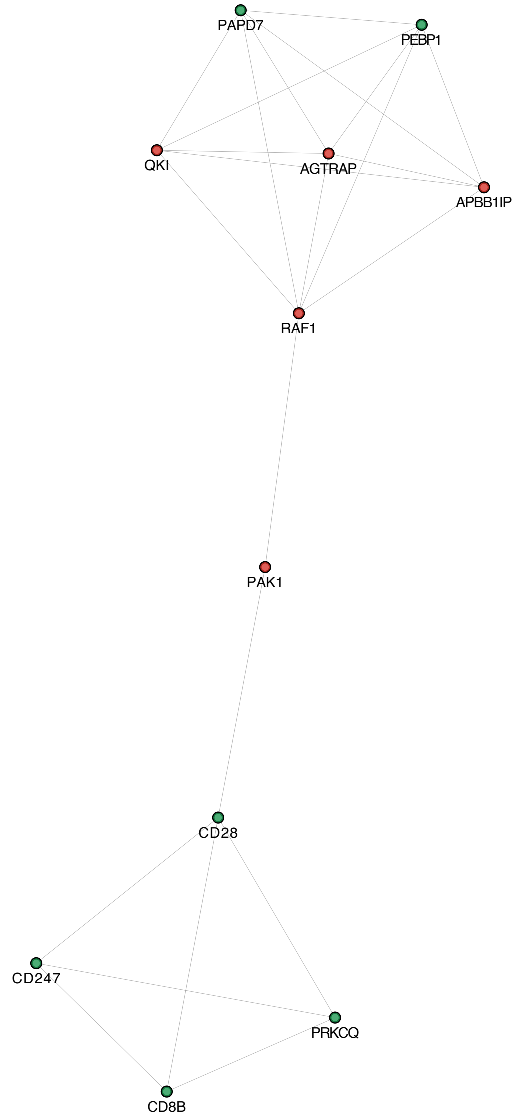

Supplementary Figure S3. MCODE analysis

O. RNA catabolic process

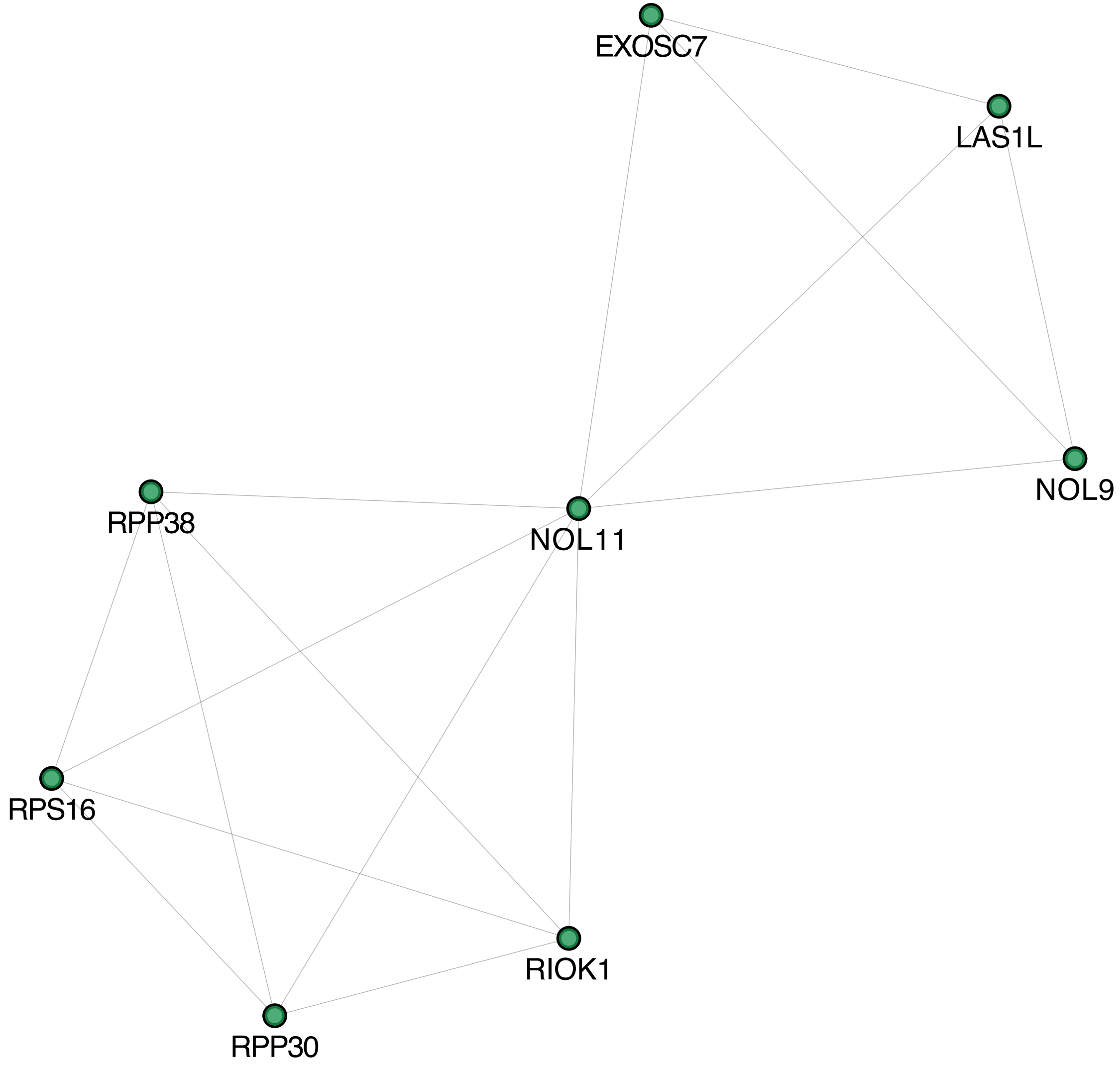

Supplementary Figure S3. MCODE analysis

P. protein localization

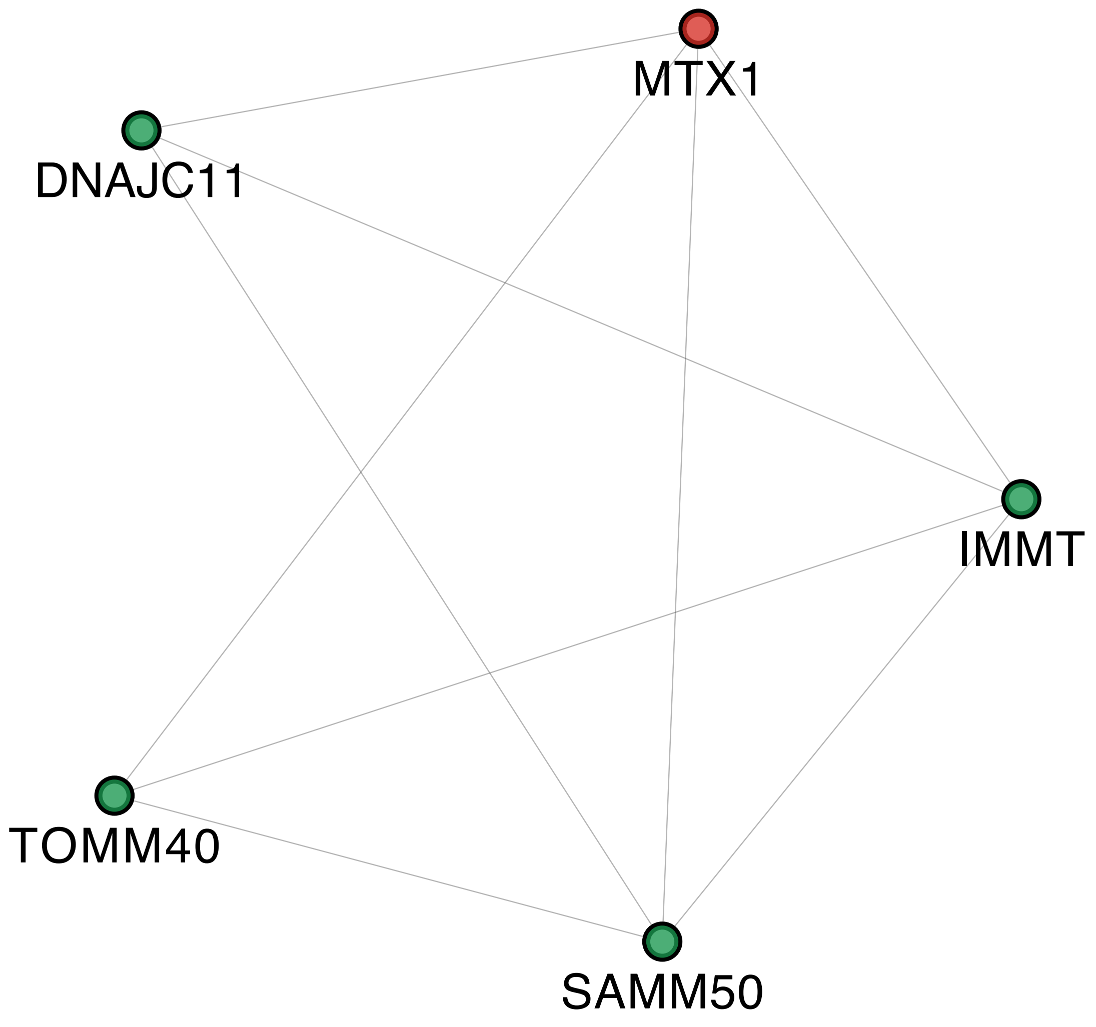

Supplementary Figure S3. MCODE analysis

Q. toll-like receptor signaling

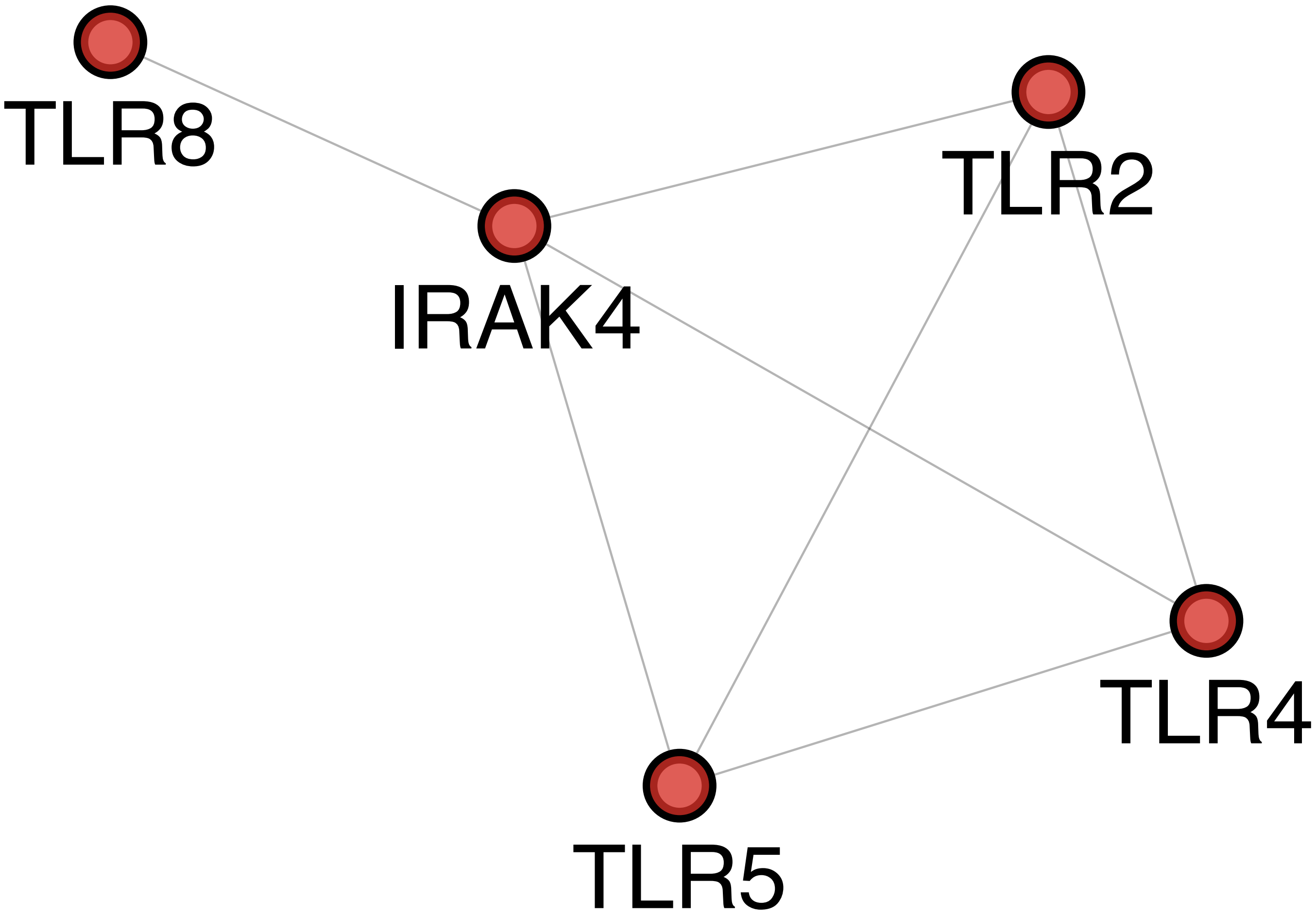

Supplementary Figure S3. MCODE analysis

R. MAPKKK cascade

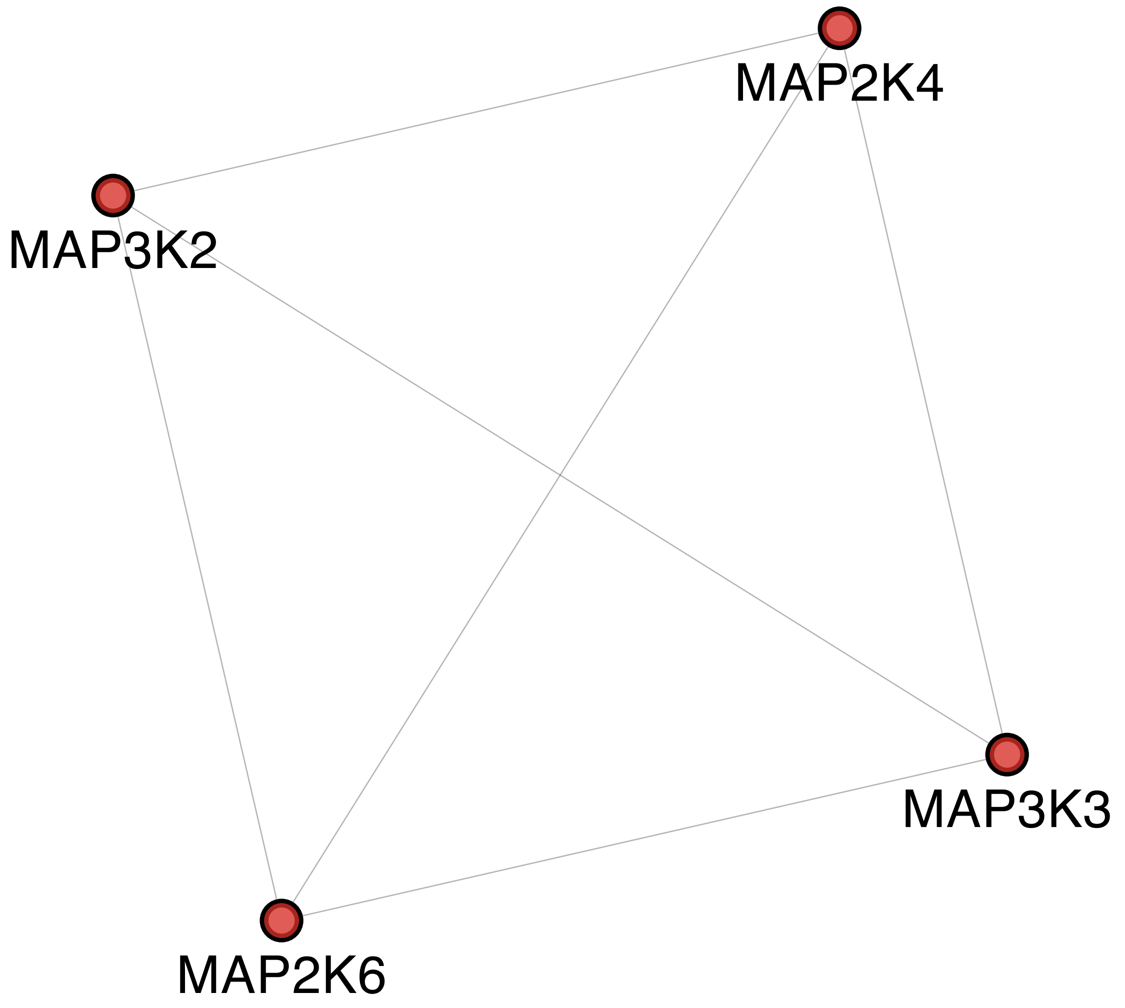

Supplement: Supplementary file 1 — Supplementary Figure S1-S3 [file 41390_2020_1347_MOESM1_ESM.pdf]
