# Supplementary material for: Gene signature of children with severe respiratory syncytial virus infection
Source: Pediatr Res. 2021 Jan 28;89(7):1664–72. doi: 10.1038/s41390-020-01347-9 (PMC8249238; doi:10.1038/s41390-020-01347-9)
Supplement: Supplementary file 3 — Supplementary Table S2 [file 41390_2020_1347_MOESM3_ESM.docx]

Supplementary Table S2. Transcriptional module analysis (TMOD)

| **Module** | **Module ID** | **Title** | **N** | **Up (%)** | **Down (%)** | **AUC** | **p-value** | **adj. p-value** |
| --- | --- | --- | --- | --- | --- | --- | --- | --- |
| Neutrophils | M37.1 | Enriched in neutrophils I | 49 | 45 (91.8) | 0 (0) | 0.9544 | 1.44x10^-37^ | 1.74x10^-35^ |
|  | M163 | Enriched in neutrophils II | 13 | 10 (76.9) | 0 (0) | 0.9098 | 6.56x10^-12^ | 1.99x10^-10^ |
|  | M11.2 | Formyl peptide receptor-mediated neutrophil response | 9 | 6 (66.7) | 0 (0) | 0.8644 | 5.58x10^-6^ | 9.95x10^-5^ |
|  | M132 | Recruitment of neutrophils | 11 | 8 (72.7) | 0 (0) | 0.8532 | 3.45x10^-6^ | 6.54x10^-5^ |
| Inflammation | M16 | TLR and inflammatory signaling | 44 | 35 (79.5) | 0 (0) | 0.9221 | 5.20x10^-24^ | 3.50x10^-22^ |
|  | M33 | Inflammatory response | 11 | 19 (36.4) | 0 (0) | 0.7614 | 3.61x10^-4^ | 4.20x10^-3^ |
|  | M25 | TLR8-BAFF network | 10 | 5 (50.0) | 0 (0) | 0.7167 | 5.53x10^-3^ | 4.03x10^-2^ |
|  | M53 | Inflammasome receptors an signaling | 12 | 4 (33.3) | 0 (0) | 0.7148 | 5.28x10^-3^ | 3.90x10^-2^ |
| Monocytes | M64 | Enriched in activated dendritic cells/monocytes | 16 | 9 (56.2) | 0 (0) | 0.8390 | 6.38x10^-6^ | 1.11x10^-4^ |
|  | M73 | Enriched in monocytes III | 12 | 5 (41.7) | 0 (0) | 0.8190 | 4.18x10^-4^ | 4.68^-3^ |
|  | M118.0 | Enriched in monocytes IV | 53 | 19 (35.8) | 0 (0) | 0.7601 | 6.05x10^-10^ | 1.67x10^-8^ |
|  | M23 | WNT receptors network (monocyte) | 11 | 7 (63.6) | 0 (0) | 0.7387 | 5.19x10^-6^ | 9.54x10^-5^ |
|  | M118.1 | Enriched in monocytes (surface) | 14 | 6 (42.9) | 0 (0) | 0.7344 | 1.27x10^-3^ | 1.22x10^-2^ |
|  | M11.0 | Enriched in monocytes II | 185 | 83 (44.9) | 0 (0) | 0.7235 | 2.04x10^-32^ | 1.77x10^-30^ |
|  | M160 | Leukocyte differentiation | 13 | 5 (38.5) | 0 (0) | 0.6770 | 5.64x10^-3^ | 4.07x10^-2^ |
|  | M4.3 | Myeloid cell enriched receptors and transporters | 29 | 9 (31.0) | 0 (0) | 0.6336 | 4.98x10^-3^ | 3.82x10^-2^ |
| Blood coagulation | M11.1 | Blood coagulation | 21 | 10 (47.6) | 0 (0) | 0.7262 | 1.02x10^-5^ | 1.67x10^-4^ |
| Platelets | M42 | Platelet activation III | 9 | 3 (33.3) | 0 (0) | 0.8791 | 4.94x10^-4^ | 5.34x10^-3^ |
| NK cells | M7.2 | Enriched in NK cells I | 45 | 3 (6.7) | 3 (6.7) | 0.5884 | 6.90x10^-3^ | 4.86x10^-2^ |
| T cells | M7.4 | T cell activation III | 15 | 0 (0) | 2 (13.3) | 0.8196 | 7.75x10^-4^ | 7.96x10^-3^ |
|  | M14 | T cell differentiation | 12 | 0 (0) | 3 (25.0) | 0.8015 | 3.82x10^-3^ | 3.09x10^-2^ |
|  | M36 | T cell surface, activation | 10 | 3 (30.0) | 0 (0) | 0.7966 | 3.98x10^-3^ | 3.16x10^-2^ |
|  | M18 | T cell differentiation via ITK and PKC | 11 | 0 (0) | 1 (9.0) | 0.7847 | 3.12x10^-3^ | 2.59x10^-2^ |
|  | M7.1 | T cell activation I | 48 | 0 (0) | 9 (18.8) | 0.7615 | 1.38x10^-6^ | 2.79x10^-5^ |
|  | M52 | T cell activation IV | 13 | 2 (15.4) | 2 (23.1) | 0.7571 | 2.40x10^-3^ | 2.11x10^-2^ |
|  | M7.0 | Enriched in T cell I | 55 | 0 (0) | 8 (14.5) | 0.7037 | 4.86x10^-5^ | 7.36x10^-4^ |

N = number of genes in the module

Up = number of upregulated genes in the module

Down = number of downregulated genes in the module

AUC = area under the curve, effect size estimate

p-value = computed using hypergeometric test

adj. p-value = p-value adjusted for multiple testing using the Benjamini-Hochberg correction
